# Immuno-metabolic dysregulation in type 2 diabetes is associated with altered neutrophil functional plasticity, mitochondrial dysfunction, and compromised responses in sepsis

**DOI:** 10.1186/s12967-026-07696-z

**Published:** 2026-01-10

**Authors:** Kailash Ganesh, Sarah Michael Gomes, Sampara Vasishta, Ganesha Poojary, Pooja Yedehalli Thimmappa, Sruthi Peesapati, Shama Prasada Kabekkodu, Sandeep Mallya, Kanive Parashiva Guruprasad, Amit Singh, Shashikiran Umakanth, Sandipan Chakraborty, Manjunath B. Joshi

**Affiliations:** 1 Department of Ageing Research, Manipal School of Life Sciences, Manipal Academy of Higher Education, Manipal, Karnataka 576104 India; 2 Department of Physiotherapy, Manipal College of Health Professions, Manipal Academy of Higher Education, Manipal, Karnataka 576104 India; 3 Center for Innovation in Molecular and Pharmaceutical Sciences (CIMPS), Dr. Reddy’s Institute of Life Sciences, University of Hyderabad Campus, Gachibowli, Hyderabad, Telangana 500046 India; 4 Department of Cell and Molecular Biology, Manipal School of Life Sciences, Manipal Academy of Higher Education, Manipal, Karnataka 576104 India; 5 Department of Bioinformatics, Manipal School of Life Sciences, Manipal Academy of Higher Education, Manipal, Karnataka 576104 India; 6 Department of Microbiology and Cell Biology, Centre for Infectious Disease Research, Indian Institute of Science, Bangalore, Karnataka 560012 India; 7 Department of Medicine, Dr. TMA Hospital, Manipal Academy of Higher Education, Udupi, Karnataka 576101 India

**Keywords:** Neutrophils, Immunometabolism, Type 2 diabetes, Sepsis, Mitochondria, Granulopoiesis

## Abstract

**Background:**

Constitutively activated neutrophil extracellular traps (NETs) have been implicated in the impeded response to infections in Type 2 Diabetes (T2D). However, immuno-metabolic factors contributing to functional plasticity of neutrophils in T2D associated infections are not known.

**Methods:**

Using both human and mice model of sepsis with either diabetic or non-diabetic background, we investigated functionally confined neutrophil subpopulations either executing phagocytosis or NETosis. An integrated analysis of cytokines regulating granulopoiesis, RNAseq of NETs forming neutrophils and metabolomics was performed. Mitochondrial function was assessed via measuring mitochondrial membrane potential, cellular oxygen consumption rate and mitophagy.

**Results:**

We identified neutrophil subpopulations either executing phagocytosis or NETosis. Proportions of these functionally restricted neutrophils are significantly altered in T2D and fail to elicit an immune response upon induction of sepsis. Integrated analysis involving cytokines, transcriptome and metabolome data revealed perturbed immunometabolic axis in T2D models majorly effecting mitochondrial metabolism. Molecular dynamic simulations indicate deformed lipid densities and disrupted inner mitochondrial membrane. T2D neutrophils showed decrease in mitochondrial membrane potential, cellular oxygen consumption rate and mitophagy. Arachidonic acid supplementation activated mitochondrial ROS and restored NETs formation in T2D upon sepsis.

**Conclusions:**

In mouse models, along with preliminary findings in human subjects, our study provides novel and correlative insights into the relationship between metabolic changes and neutrophil dysfunction in T2D-associated sepsis, exploring immuno-metabolism as a therapeutic target to improve neutrophil function.

**Supplementary Information:**

The online version contains supplementary material available at 10.1186/s12967-026-07696-z.

## Background

Bidirectional activation of the immune system and metabolism forms an immuno-metabolic axis that intricately regulates the differentiation, generation, activation, and functions of immune cells. Hence, nutrients and metabolic flux play important roles in the regulating effector functions of immune cells during both the steady state and infection. In response to pathological stimuli such as infectious agents or sterile inflammatory mediators, immune cells adapt to their energy demands and specific metabolic substrates [1]. Therefore, alterations in either inflammatory or metabolic signalling often reprogram metabolism in immune cells and influence their homeostatic functions. Consequently, these changes contribute to the pathogenesis of several diseases characterized by chronic inflammation such as Type 2 Diabetes (T2D) [2].

T2D is associated with perturbations in immuno-metabolic signalling characterized by increased immune cell infiltration, chronic low-grade inflammation, elevated oxidative stress and metabolic impairments [3]. A nutrient rich state in T2D with hyperglycaemia, excess lipids and elevated branched chain amino acids influences both systemic and cellular metabolism and impacts the functions of immune cells including neutrophils. Experimental, pre-clinical and clinical studies in T2D have demonstrated neutrophil dysfunction with decreased levels of reactive oxygen species (ROS), impaired chemotaxis, reduced phagocytic properties and simultaneously constitutive production of extracellular traps [4–7]. These functional alterations in neutrophils have been attributed to the manifestation of T2D associated infections [8], delayed wound healing [9] and vasculopathies [10]. Previously, we have shown that hyperglycaemia induces constitutive NETs formation and positively correlates with fasting and post-prandial glucose in T2D subjects [11, 12]. Untargeted metabolomics analysis of neutrophils from T2D subjects revealed perturbed polyol pathway and glutathione metabolism contributing to altered redox state. Nicotinamide Adenine Dinucleotide Phosphate (Reduced form) (NADPH) is an absolute requirement for three independent pathways (a) formation of 1-anhydrosorbitol *via* aldose reductase under excess glucose, (b) glutathione synthesis, and (c) glucose induced NETs formation. During T2D, we observed competition for NADPH between these biochemical processes, which led to the inability of neutrophils to produce NETs in response to Lipopolysaccharide (LPS) [6]. Hyperglycaemia primes neutrophils to form NETs in Padi4 dependent manner, which subsequently resulted in delayed diabetic wound healing [9]. Impaired neutrophil migration to airways in response to LPS was observed in T2D model of Goto-Kakizaki (GK) rats. GK rats exhibited reduced levels of chemokines and cytokines such as Interleukin (IL)-1β and Tumor Necrosis Factor-alpha (TNF-α) concentration and decreased expression of Lymphocyte Function-associated Antigen-1 (LFA-1) and Intercellular Adhesion Molecule-2 (ICAM-2) on neutrophils [13]. Higher concentrations of intracellular calcium and reduced Adenosine Triphosphate (ATP) levels associated with hyperglycaemia inhibited the phagocytic ability of neutrophils, which was restored upon glycaemic control [14].

Neutrophil homeostasis depends on a balance between granulopoiesis and the clearance of aged and apoptotic neutrophils by efferocytosis [15]. During steady-state granulopoiesis, a constant supply of neutrophils is maintained by Granulocyte Colony-Stimulating Factor (G-CSF) levels [16]. However, in response to infections, the demand for neutrophils increases, leading to the activation of emergency granulopoiesis regulated by CCAAT/enhancer-binding protein beta (CEBP-β). During infections, Pathogen-Associated Molecular Patterns (PAMPs) and Damage-Associated Molecular Patterns (DAMPs) which are recognised by other immune cells and endothelial cells, produce inflammatory mediators (IL-6, IL-1β, IL-3, Fms-like Tyrosine Kinase 3 (FLT3), G-CSF, Granulocyte-Macrophage Colony-Stimulating Factor (GM-CSF), IL-17 and TNF-α), which stimulate the process of emergency granulopoiesis to induce neutrophil production and trafficking into the bloodstream [17].

T2D individuals exhibit immune dysfunction without compromising for neutrophil counts, both under steady state and infections. This indicates dysregulated metabolism in T2D characterized by a nutrient excess state which may alter only functional plasticity of neutrophils without modulating their counts. Hence, spatio-temporal metabolic regulation of neutrophil effector functions including energy demands, adapting for specific nutrient substrates and bioenergetics required for eliminating pathogens may vary between healthy and T2D individuals. Therefore, understanding the crosstalk between inflammatory mediators and metabolism in T2D during steady state and emergency granulopoiesis may facilitate the identification of new mechanisms and therapeutic targets for the management of T2D-associated infections.

## Materials and methods

### Ethical clearance

We obtained the ethical clearance from institutional ethics committee, Manipal Academy of Higher Education, Manipal (472/2020). Approximately 5 ml of peripheral blood was collected, with prior written informed consent, from healthy and diabetic individuals with or without sepsis at TMA Pai Hospital, Udupi, India, for neutrophil isolation. Hba1c level greater than 6.5% was considered as T2D for the participation. Bacterial sepsis subjects confirmed via culture technique and CRP levels were considered for the participation. All procedures were performed in accordance with national guidelines and regulations.

### Establishment of the T2D and sepsis mouse models

Upon obtaining ethical clearance from the institutional animal ethics committee, Manipal Academy of Higher Education, Manipal (IAEC/KMC/07/2020), we established mouse models for T2D and sepsis. Both male and female mice (6–8-week-old C57BL/6) were housed, and 12 h day/dark cycle was maintained. The animals were grouped into (a) Control (*n* = 24), (b) High fat T2D (*n* = 24). The diabetes phenotype was induced upon feeding the animals with high fat diet (HFD) (60% kcal from fat) (Star Enterprises, India) for 20 weeks. Mice were monitored for gain in weight and plasma glucose levels. After confirming elevated glucose levels, half of the animals were re-grouped and were subjected to induce sepsis by cecal ligation and puncture (CLP) upon perforation of the cecum, which allows the release of fecal material into the peritoneal cavity to generate an exacerbated immune response induced by polymicrobial infection. The mice were monitored and sacrificed according to humane endpoints. Sepsis was confirmed by platelet counts, White Blood Cells (WBC) counts, bacterial growth in the blood, cytokine measurement and assessed for survival curve. Animals were randomly assigned to experimental groups, and investigators were blinded during sample processing and analysis. Blood collected through the retro-orbital puncture method was subjected to plasma extraction and frozen at -80 °C until further use.

### Isolation of rodent neutrophils

Neutrophils were extracted from peripheral blood and bone marrow (BM) following a depletion strategy using magnetic bead-based separation methods (BioLegend, UK) as described by the manufacturer. In brief, blood and BM collected from the mouse were subjected to density gradient centrifugation using Ficoll ^®^ Paque Plus (Cytiva, USA). After discarding mononuclear cells, PMNs and RBCs were treated with RBC lysis buffer at a ratio of 1:10 to remove the RBCs. Neutrophils were enriched using an antibody cocktail mixture and magnetic beads (Mojosort mouse neutrophil isolation kit, BioLegend, UK). The cell mixture was placed in a magnetic field to sort out other blood cells (Negative selection). Cell viability and counting were performed using Trypan blue staining. The isolated neutrophils were confirmed by flow cytometry using a PE-conjugated Rat Anti-Mouse LY-6G antibody.

### Isolation of human neutrophils

Neutrophils were isolated using a well-established Ficoll-dextran method [12]. Briefly, peripheral blood was layered on Ficol ^®^ paque Plus and centrifuged at 2000 rpm for 20 min. The red pellet at the bottom was diluted with HBSS buffer and incubated with 500µL of dextran for one hour. Clear phase was collected and centrifuged at 1800 RPM for 10 min. The pellet obtained was treated with RBC lysis buffer to enrich the neutrophils. Cell viability and counting were measured using Trypan blue staining.

### Identification and characterization of neutrophil subpopulations

Neutrophils isolated from peripheral blood and BM were tagged with neutrophil-specific antibody (PE-Ly6G-mice; PE-CD16B – Human (BD Biosciences, USA)) and treated with *E. coli* bioparticles tagged with Alexa Fluor 488 at a ratio of 1:10 and incubated for 15 min. Neutrophils were stained with anti-citrullinated histone H3 primary antibody (1 µg/mL) (Abcam, Cambridge, UK) for 30 min and washed with PBS. Followed by secondary antibody Alexa Fluor 633 (1 µg/mL) (Thermofisher Scientific, USA) for 30 min and washed with PBS. Further cells were processed for acquisition (10000 cells) in flow cytometry to assess the NETs formation and phagocytosis. As a first step, the neutrophil population was considered based on Ly6G (mouse) or CD16B (human) expression. Further, a gating strategy was applied on a quadrant basis. Q1 indicated neutrophils positive for NETs formation (Anti-citrulline histone H3); Q4 represented neutrophils performing phagocytosis alone (*E. coli* bioparticles); Q2 population signified cells performing both phagocytosis and NETs; cells in Q3 region were negative for both the stains (Inactive neutrophils).

### RNAseq analysis

NETs-forming neutrophils were separated from non-NET-forming neutrophils derived from bone marrow using a magnetic bead-based strategy. Briefly, neutrophils isolated from control and T2D animals were treated with lipopolysaccharide (LPS-2 µg/mL) for 30 min to induce the NETs formation. Further cells were treated with a mixture of Pierce protein A magnetic beads and anti-citrullinated histone H3 antibody (1:3). After incubation for 30 min, the cell mixture was placed in a magnetic field to separate NETs forming neutrophils and non-NETs forming neutrophils. Further to confirm the separation, flow cytometry acquisition was performed only using a secondary antibody against citrullinated histone.

RNA was extracted from NETs - forming and non-NETs-forming subpopulations using the previously established TRIZOL method. Further, we pooled the RNA of the respective subpopulations from control and T2D animals (*n* = 3), and quantitation was performed using a NanoDrop for OD at 260/280 and a Qubit spectrophotometer (Thermofisher, USA). After the depletion of ribosomal RNA, the remaining RNA was converted to cDNA. Further library was prepared and sequenced on a NovaSeq 6000 (Next Generation Sequencing facility, CSIR-Centre for Cellular and Molecular Biology, India).

### Bioinformatic analysis

The fastq files obtained following the transcriptomic run of the mice BM neutrophil subpopulations were analysed using nf-core/rnaseq pipeline version 3.10.1 on Nextflow version 22.10.4 [18, 19]. Raw read quality was evaluated using FastQC and summary reports were compiled using MultiQC [20, 21]. The reads were aligned to the mouse reference genome (GRCm38) using the STAR aligner and the gene-level counts were obtained using featureCounts tool [22, 23]. EdgeR, a R Bioconductor package, was used to carry out the differential expression (DE) analysis between groups [24]. Genes were considered significantly upregulated or downregulated based on the defined criteria of p-value < = 0.05 and fold change of > = 1.5 and <= -1.5 respectively.

### Analysis of NETs components

Neutrophils were stained with an anti-citrullinated histone H3 antibody and processed for acquisition in flow cytometry to assess the NETs formation. The cell-free DNA in the plasma was quantified by Sytox Green (Thermo-Fischer’s, USA) DNA staining and fluorimetry technique (Varioskan, Thermo Fischer). Neutrophil elastase was quantified by ELISA (Abcam, UK).

### Analysis of inflammatory mediators associated with granulopoiesis

Inflammatory cytokines regulating granulopoiesis including C-C Motif Ligand 2 (CCL2), G-CSF, IFN-γ, IL-6, TNF-α, GM-CSF, IL-1β, C-X-C chemokine (CXCL2) and IL-17 were measured using Mouse Luminex Discovery Assay 9 PLEX according to the manufacturer’s protocol, which was performed on a Magpix Luminex ELISA system (Thermo Fischer, USA).

### Global metabolomics analysis

Untargeted metabolomics analysis was performed using ESI-QTOF-LC/MS (Agilent 6520, Santa Clara, USA) coupled to an Agilent 1200 liquid chromatography system with a spectrometer. Briefly, 100 µL of plasma was mixed with 200 µL of ice-cold methanol, allowing maximum recovery of small molecules. The mixture was further centrifuged at 13,200 rpm for 15 min. The extract obtained was dried under vacuum and the obtained residue was reconstituted in 30 µL of water: acetonitrile (95:5) containing 0.1% formic acid. A 5 µL aliquot was injected onto a 4.6 × 150 mm Phenomenex Aqua-C18 5 μm particle column maintained at 25 °C. The plasma metabolites were gradient-eluted at 400 µL/min using mobile phase A:0.1% formic acid in water and mobile phase B: 0.1% formic acid in 90% acetonitrile (2% to 98% B in 25 min, 98% B for 10 min and equilibrated to 2% B for 10 min). Electrospray ionization (ESI) was used in positive mode with spray voltage ion injection at 3.5 kV. The mass analyzer was scanned over a range of 50–1700 m/z. The MS interface capillary was maintained at 350 °C, with a sheath flow of 8 L/min. Agilent Mass Hunter Workstation Data Acquisition Software B.02.01 (Agilent Technologies, Santa Clara, CA, USA) was used for instrument control and data acquisition. The raw data obtained were converted into mzml file format using msconvert. Further, XCMS database was used to obtain M/Z values of each compound along with the corresponding abundance values.

### Extracellular flux analysis

All the assays were carried out using Agilent Seahorse XF8 cell culture microplates and coated with poly-L-lysine (25 µg/mL) for 3 h before the adhesion of the cells. Pre-coated microplates were seeded with 40,000 cells/well (mitochondrial respiration and oxidative burst) in 50µL XF media. To account for background correction, 50 µL of RPMI assay media was added to background control wells A and H (no cells). The XF8 plates were centrifuged at 200*g for 1 min with a break and acceleration set at 0. An additional 130 µL of assay media was gently added (total volume = 180 µL) to each well and incubated at 37 °C without CO2 for 30 min prior to XF assay. For the mitochondrial respiration, 20 µL, 22 µL and 25 µL respectively of each inhibitor were loaded into injection ports A–C of the sensor cartridge in the following order: Port A − 1.5 µM oligomycin; Port B − 0.5 µM FCCP (carbonyl cyanide-4-(trifluoromethoxy)phenylhydrazone) and Port C − 0.5 µM rotenone/antimycin A. All compounds were prepared in XF media.

### Mitochondrial imaging and reactive oxygen species (ROS) measurement

Neutrophils isolated from different animal models and human peripheral blood were treated with MitoTracker Red (Thermofisher Scientific, USA) for 20 min. Further, the cells were fixed in chamber slides and stained with DAPI to stain the DNA. Images were acquired using a confocal microscope (Leica Microsystems, Germany). To measure the mitochondrial ROS formed, neutrophils were treated with MitoSox Red and processed for flow cytometry.

### Assessment of mitophagy

Mitophagy in neutrophils was examined using the mitophagy detection kit (Dojindo, Japan). Briefly, freshly isolated neutrophils were incubated at 37 °C for 30 min with 100 nM Mtphagy Dye. The cells were washed twice and treated with high glucose (10 mM) for one hour and then subjected to flow cytometry. Mitophagy dye was excited using a 488 nm laser and detected at 695 nm. A subset of cells showing high intensity of Mtphagy dye due to alteration in pH, indicating fusion of mitochondria and lysosomes, was considered for further analysis.

### Molecular dynamics simulations of the mitochondrial membrane in different disease conditions

The coarse-grained mitochondrial lipid bilayers were built using the *insane* [25] utility. We considered four conditions: control, T2D, Sepsis, and T2D with sepsis. The initial membranes were modeled within a stimulation box with ~ 50 × 50 × 20 nm^3^ dimensions. The membrane composition of control systems was based on a previous study [26]. The membrane lipids were represented using coarse-grained Martini 2.2 parameters [27]. Fatty acids (18:1 and 18:2) parameters were generated from the template of C18:0 with modifications on bead type and angle parameters to account for the degree of unsaturation. Lysophospatidylethanolamine parameters were generated using the *lipidmartini-itp-v05.py* script [25]. The simulations were performed using the GROMACS 2020.6 [28] simulation package. The simulation box was solvated with coarse-grained water (W). Na^+^ and Cl^−^ ions were added to neutralize the bilayer components and maintain a salt concentration of 150 mM. All the systems were energy minimized with the steepest descent algorithm. Each system was then subjected to a series of equilibrium simulations, starting with a 5 ns simulation in the NPT ensemble with a 10 fs time step, 10 ns of simulation in the NVT ensemble with a 20 fs time step, followed by 10 ns of simulation with a 20 fs time step in the NPT ensemble. The final production runs were carried on for 2 µs at 300 K in an NPT ensemble with a 20 fs time step. Each of the systems was simulated in triplicate with periodic boundary conditions. The system components were coupled using a v-rescale [29] thermostat to maintain the simulation temperature of 300 K for equilibration and production runs with a coupling time constant of 1 ps. A semi-isotropic pressure coupling scheme with a Berendsen thermostat was used for equilibration simulations with a time constant of 4 ps to maintain pressure at 1 bar, while Parinello-Rahman barostat [30] was used during the production runs with a coupling time constant of 12 ps. Bond lengths were constrained using the LINCS algorithm.

### Immunoblotting

Neutrophils isolated from the peripheral blood of healthy volunteers were stimulated with 1 µM and 10 µM of arachidonic acid for 30 min to induce the formation of NETs. Cells were collected by centrifuging at 1800 RPM for 10 min and lysed using RIPA buffer. Protein concentrations were determined by Lowry’s method. On a 10% denaturing polyacrylamide gel, equal concentration of proteins were loaded and transferred onto nitrocellulose membranes. Blots were further probed with primary antibody: Anti-p44/42 ERK (Thr202/Tyr204) and Anti-ERK (Cell Signaling Technology, Massachusetts, USA), followed by secondary antibodies conjugated with horseradish peroxidase (Thermo Fisher, US). Immunoblots were developed using an enhanced chemiluminescence kit (ECL) (Pierce, Thermo Fisher Scientific, MA, USA) and imaged in iBRIGHT imaging system (Thermofisher Scientific, USA). Images of immunoblots were processed using ImageJ software (NIH, USA) for densitometric analysis.

### Statistical and bioinformatics analysis

The metabolite dataset was analysed via tools such as MetaboAnalyst and SPSS. High- throughput data were subjected to multi-variate analysis, such as principal component analysis and Partial Least Squares Discriminant Analysis (PLS-DA) to understand the stratification of the data. Further annotation of the obtained compounds was performed using the HMDB database. Two-way ANOVA and t-test were conducted to assess significant modulation of metabolites between and among groups. Pathway analysis was performed using KEGG and MetaboAnalyst 5.0 tools. Metabolites found were correlated using Pearson’s correlation analysis between metabolites obtained from all the groups and inflammatory mediators using ORIGIN 10.0.0.154.

## Results

### Neutrophils exhibit functional heterogeneity with distinct subpopulations confined to phagocytosis and NETs formation in the steady state

**Fig. 1:**
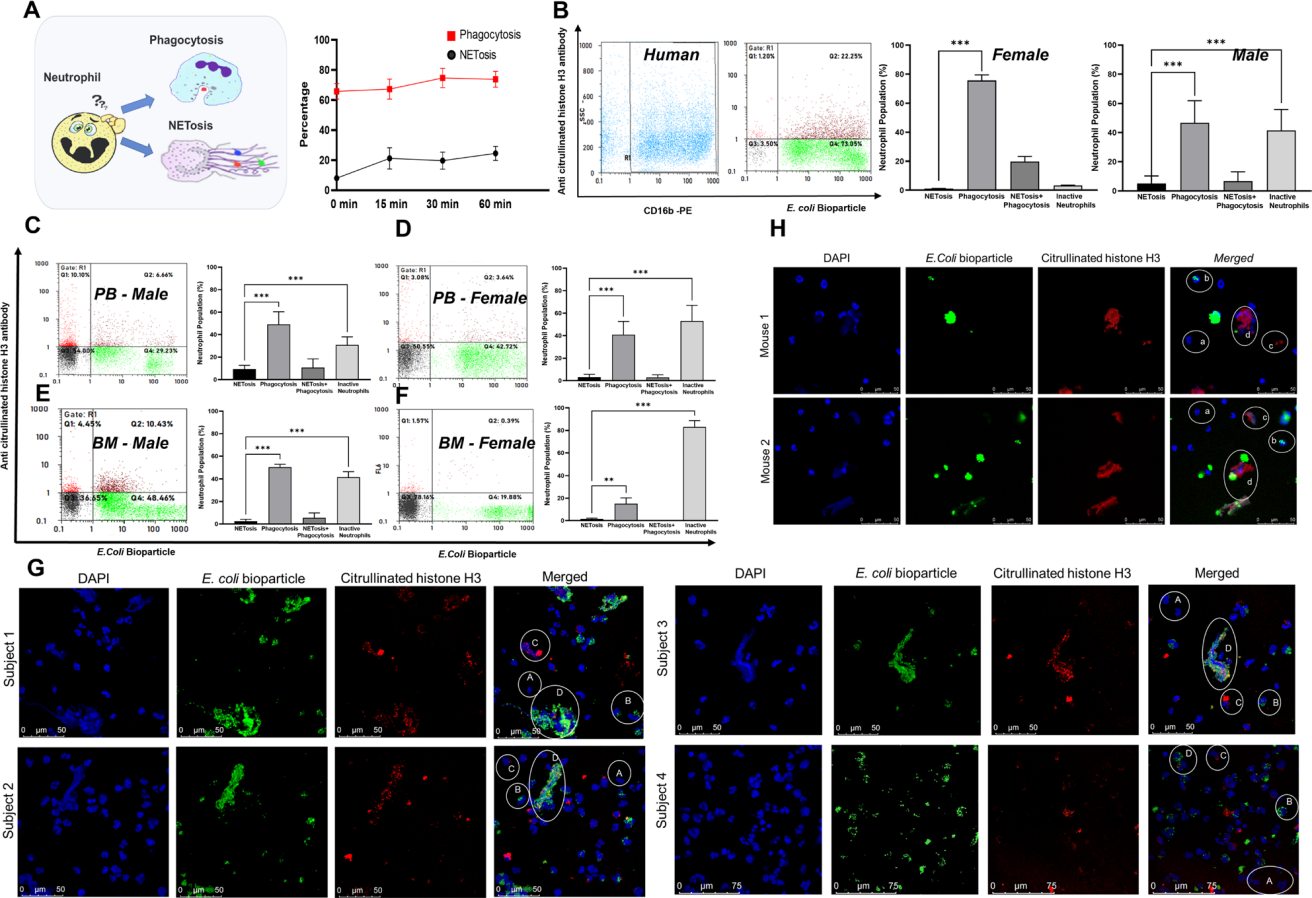
Neutrophils display functional heterogeneity. Freshly isolated neutrophils were treated with *E. coli* bioparticles in a ratio of 1:10 along with anti- citrullinated histone H3 antibody for the indicated time points followed by incubation with the secondary antibody conjugated with AlexaFluor 633. The cells were washed with PBS and processed using flow cytometry. **A**. Kinetics of cells undergoing phagocytosis and NETs formation are represented as percentage. Neutrophils isolated from human peripheral blood (**B**) (*n* = 6; Male = 3; Female = 3) and mouse peripheral blood (**C** & **D**) (*n* = 12; Male = 6; Female = 6) and Bone marrow (**E** & **F**) (*n* = 6; Male = 3; female = 3) were treated as above and the percentages of cells undergoing phagocytosis and NETosis were determined by flow cytometry. Dot plots and cumulative bar graphs showing the abundance of functionally confined neutrophils are provided. Statistically significant differences are denoted by asterisks * = *p* < 0.01, ** = *p* < 0.001, *** = *p* < 0.0001. Confocal imaging of neutrophils from humans; *n* = 4 **(G)** and mice *n* = 3 **(H)** displaying their ability to undergo either phagocytosis *E. coli* bioparticle (AlexaFluor 488), or NETosis (Cit-Histone H3; AlexaFluor 633) are represented. The nuclei were stained with DAPI. Subpopulations of neutrophils are denoted by circles. A, a. Inactive neutrophil, B,b. Phagocytosis, C,c. NETosis, D,d. Phagocytosis + NETosis

We first evaluated the kinetics of phagocytosis and NETosis in mouse peripheral blood neutrophils. Upon exposure to *E. coli* bioparticles, neutrophils initiated phagocytosis instantly, reaching 65% in 0 min and increasing to 70% by 60 min. Interestingly, only 5–6% of the neutrophils showed basal citrullinated histone levels which increased to 14–18% by 60 min (Fig. 1A). This indicates phagocytosis is followed by NETosis.

Further experiments aimed to identify whether functionally distinct neutrophil subpopulations exist in the steady state and whether the proportions of these subpopulations vary under pathological conditions. Hence, a series of flow cytometry-based analyses was performed to examine the functional heterogeneity of neutrophils in response to *E. coli* bioparticles to assess their ability to carry out phagocytosis or NETs formation. Bioparticle internalization and citrullinated histone levels served as an indicator for phagocytosis and NETosis respectively. Flow cytometry analysis of peripheral blood neutrophils from male mice incubated with *E. coli* bioparticle at 37 °C revealed four distinct subpopulations (a) positive for only citrullinated histones representing NETosis (9.33 *±* 3.38%), (b) internalized *E. coli* bioparticles indicating phagocytosis (49.05 *±* 11.34%), (c) double positive cells with the ability to perform both phagocytosis and NETosis (10.78 *±* 7.66%) and (d) inactive neutrophils which were unable to execute neither phagocytosis nor NETosis (30.84 *±* 7.19%) (Fig. 1C). However, neutrophils incubated with bacterial bioparticles at 4 °C (negative control) only showed the ability to undergo phagocytosis but not NETosis (Fig. S1A). Taken together, these findings indicate the existence of neutrophil subpopulations confined to perform either phagocytosis or NETosis under normal physiological conditions.

Further, we examined the distribution of functionally heterogeneous neutrophils in different tissues, gender and organisms. Like male mice, neutrophils from female animals also exhibited functionally restricted subpopulation where 3.07 *±* 2.51% formed NETs, 40.86 *±* 11.84% drove phagocytosis, 3.02 *±* 2.51% exhibited the ability to perform both NETosis and phagocytosis and over 52.99 *±* 14.01% were not involved in either NETosis or phagocytosis (Fig. 1D). The functionally heterogeneous properties of neutrophils were also evident in bone marrow derived neutrophils from both male and female animals. However, these neutrophils showed reduced ability to form NETs compared to that of peripheral blood neutrophils in both male (2.47 *±* 1.73% vs. 9.33 *±* 3.38%) and female mice (1.66 *±* 0.53% vs. 3.07 *±* 2.51%) (Fig. 1E and F). Interestingly, we observed gender differences in forming NETs where female mice showed significantly reduced response to form NETs compared to male animals in both bone marrow and blood-derived neutrophils (Fig. S2A). Further, bone marrow derived neutrophils from female animals were abundantly inactive compared to male animals (Fig. 1E and F).

Subsequently, we asked whether neutrophils display similar subsets in healthy human subjects. Both male (*n* = 3) and female (*n* = 3) human individuals displayed functional heterogeneity of neutrophils. In response to *E. coli* bioparticles, neutrophils isolated from male individuals displayed 46.67 *±* 15.19% of phagocytic neutrophils, less than 5% of NETs forming neutrophils, 6.6 *±* 6.4% were double positive for phagocytosis and NETs, whereas 41.51 *±* 14.33% represented inactive neutrophils. In female human subjects, only 1.11 *±* 0.12% neutrophils exhibited the ability to form NETs, and a significant proportion was confined to phagocytosis 75.75 *±* 3.81%, 19.78 *±* 3.49% showed both phagocytic + NETs forming neutrophils and 3.37 *±* 0.18% of inactive neutrophils (Fig. 1B). On similar lines with findings in animal models, neutrophils derived from human female individuals also displayed reduced ability to form NETs. Confocal imaging of neutrophils stimulated with *E. coli* bioparticles also revealed functionally heterogeneous cells performing phagocytosis or NETosis in both human and animal models (Fig. 1G and H). These results confirmed the existence of functionally restricted neutrophil subpopulations in mouse and human models in peripheral blood and bone marrow.

### Proportions of neutrophil subpopulation significantly alter in pathological conditions

**Fig. 2:**
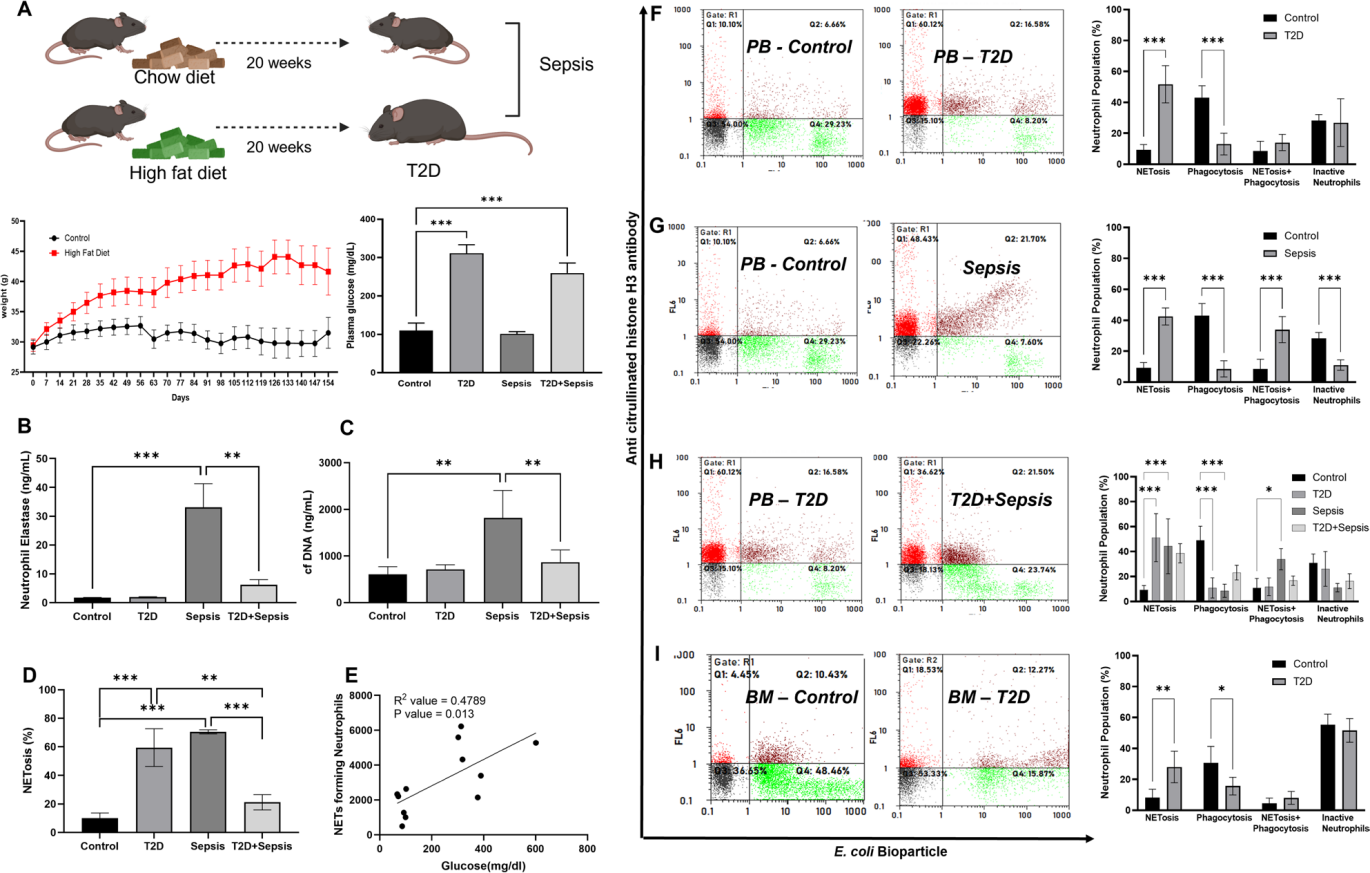
T2D and sepsis are associated with changes in the proportions of neutrophil subpopulation with confined functions. **A.** Experimental design to develop and validate animal models of T2D with or without sepsis is shown. Changes in body weight, plasma glucose level and cholesterol levels SF3A indicated development of T2D. Neutrophil elastase (**B**) and cell free DNA (**C**) was measured in mouse plasma of control (*n* = 6); T2D (*n* = 6); Sepsis (*n* = 6) and T2D + Sepsis (*n* = 6). **D**. Neutrophils isolated from peripheral blood were stained for citrullinated histones using flow cytometry and fasting plasma glucose levels were measured by autoanalyzer. **E.** The data represents the correlation between the abundance of neutrophils forming NETs and plasma glucose R^2^ = 0.4789, *p* < 0.01. **F**,** G**,** H**,** I.** Sub-populations of neutrophils undergoing either phagocytosis or NETosis or both in control and T2D models with or without sepsis were determined using flow cytometry. Representative flowcytometry dot plots and cumulative bar graphs displaying functional heterogeneity of neutrophils isolated from blood and bone marrow from different study groups (*n* = 6 in each group) are provided. Statistical significance was obtained by performing two-way ANOVA followed by followed by Tukey’s multiple comparison test to control the family-wise error rate. Adjusted p-values are represented as asterisk. (* = *p* < 0.01, ** = *p* < 0.001, *** = *p* < 0.0001)

As our earlier studies have demonstrated constitutive NETs formation leading to reduced response to infections in T2D [6, 11, 12], we examined proportions of functionally-confined neutrophils in healthy and HFD induced T2D models with or without sepsis. HFD fed mice showed significantly higher weight gain (Male – 51.63 ± 9.69 g) compared to the mice on standard chow feed (Male – 31.5 ± 7.21 g) displaying the characteristics of obesity after 20 weeks and showed significant increase in the levels of fasting plasma glucose (Male – 381.24 ± 47.55 mg/dL) compared to the control group (Male – 128.67 ± 5.15 mg/dL) and Total cholesterol (HFD – 137.04 ± 10.83 mg/dL; Control 66.05 ± 17.37 mg/dL) (SF3A) confirming the induction of T2D in mice (Fig. 2A).

T2D mouse models showed a significant decrease in neutrophil elastase (*p* < 0.001) and cell free DNA (*p* < 0.001) upon induction of sepsis (Fig. 2B and C). Further, T2D animals showed significantly elevated levels of constitutive NETosis (59.92 *±* 10.79% vs. Control-10.09 *±* 2.94%). Sepsis induction also showed an increase in NETs levels. However, T2D animals failed to mount NETosis in response to sepsis. (Fig. 2D). Pearson’s correlation analysis revealed a positive association between glucose levels and the magnitude of NETs formation (*p* = 0.013; R^2^ = 0.4789) in T2D animals (Fig. 2E). These data reaffirmed our earlier findings in humans where we demonstrated reduced response of neutrophils from T2D individuals to LPS to form NETs.

Upon stimulation with *E. coli* bioparticles, T2D neutrophils exhibited drastic alterations in the proportions of functional subsets. NETs forming neutrophils remained at constitutive levels (51.10 *±* 19.25% vs. 59.92 + 10.79%) and did not form NETs further in response to *E.coli* (Fig. 2D and F). On the other hand, phagocytic neutrophils were significantly compromised in T2D models (10.89 *±* 8.13%) against control mice (49.05 *±* 11.34%). An abundance of double positive cells for NETs and phagocytosis and inactive neutrophils remained similar between control and T2D mice (Fig. 2F).

To determine functional differences and plasticity of neutrophils in non-diabetic and diabetic animals, we induced sepsis in control and T2D animals (Fig. S4A). Caecal ligation puncture survival surgery resulted in a significant reduction in the platelets and WBC counts (Fig. S5A), elevated bacterial load (Fig. S6A), cytokine levels (Fig. S7A) and survival curve (SF8A) confirmed the successful induction of sepsis. Neutrophils isolated post 24 h of sepsis induction presented a substantial increase in NETs (36.78 *±* 13.54%), and reduced phagocytosis. A marked increase was observed in the proportions of neutrophils with the ability to perform both phagocytosis and NETs (33.94 ± 8.45%) compared to controls. Interestingly, abundance of inactive neutrophils was decreased (12.25 *±* 5.8% vs. 30.84 *±* 7.19% in baseline) (Fig. 2G). However, when sepsis was induced in T2D background, animals failed to increase their ability to form NETs and phagocytosis (Fig. 2H). Further, the transition of inactive neutrophils to the active form was not observed in sepsis with T2D background (Fig. 2H). The proportions of NETs forming and phagocytic neutrophils also did not vary in T2D animals after the induction of sepsis (Fig. 2H).

Subsequently, we examined functionally restricted subpopulations in bone marrow derived neutrophils in control and T2D models with or without sepsis. In T2D animals, NETs forming neutrophils were significantly elevated (27.95 *±* 10.21%) compared with those in control (2.47 *±* 1.73%), phagocytic neutrophils were greatly reduced (15.61 *±* 5.70%) and no changes were observed in double positive cells for phagocytosis and NETs and inactive cells (Fig. 2I). Constitutively activated BM neutrophils from T2D animals also showed a similar weaker response to LPS to form NETs (Fig. S9A). Taken together, these results suggest that proportions of functionally confined neutrophils are altered in T2D animals and might therefore fail to elicit an immune response in sepsis.

### Neutrophils with the ability to form NETs show distinct expression of genes than those that do not form NETs and vary in T2D models

**Fig. 3:**
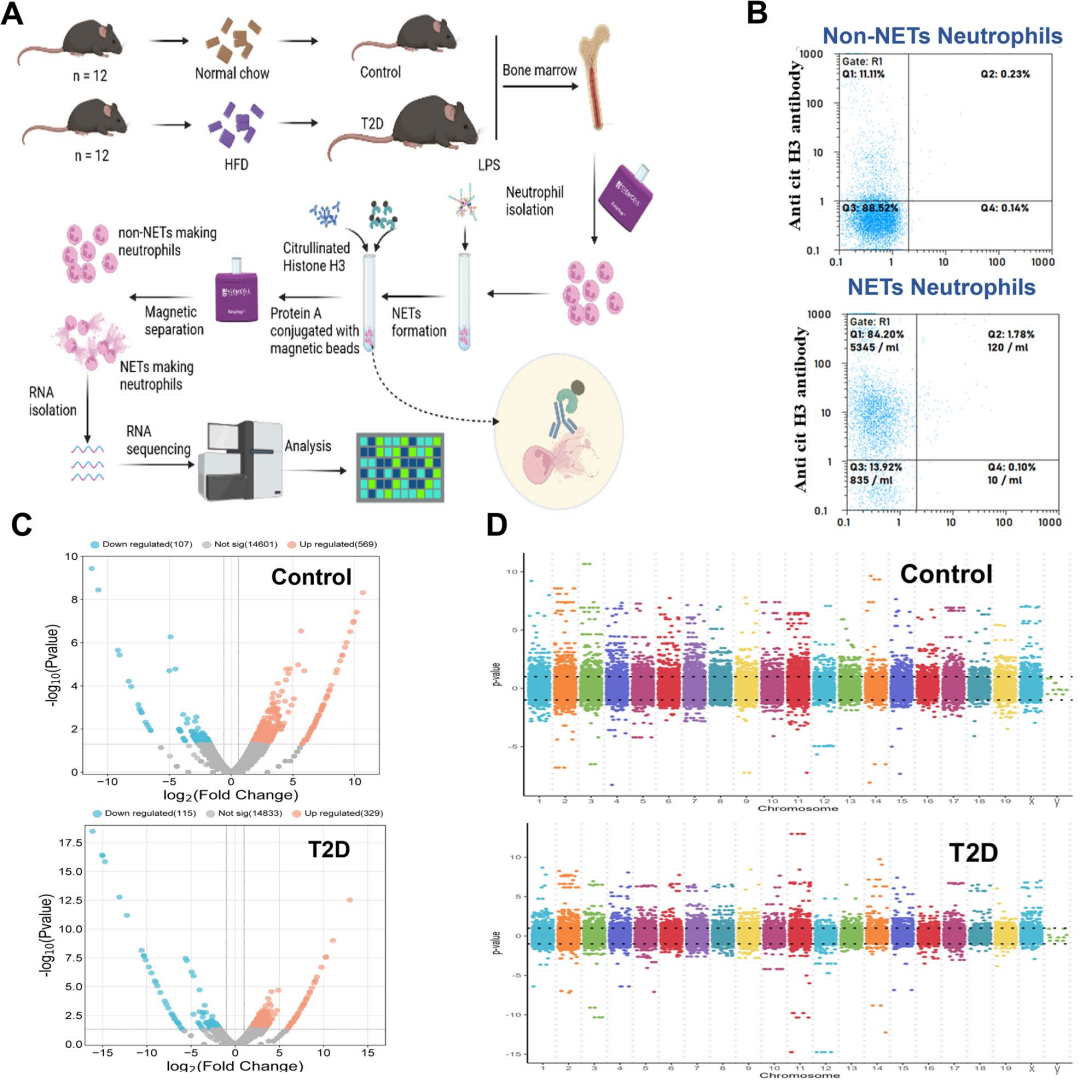
The neutrophil subpopulation with the ability to form NETs shows a distinct transcriptome (NF: NETs forming neutrophils and NNF: Non-NETs forming neutrophils; C: Control and T: T2D). **(A)** Experimental design to separate neutrophils based on their ability to form NETs. Neutrophils isolated from the bone marrow of control (*n* = 3) and T2D mice (*n* = 3) were treated with LPS for 30 min to induce NETs formation and followed by incubation with anti-citrullinated histone antibodies. Further, NETs forming neutrophils (NF) displaying citrullinated histones were separated from non-NETs forming neutrophils (NNF) using a magnetic bead-based strategy. RNA isolated from these two populations was subjected to total RNA sequencing. **(B)** Flow cytometry validation for separated neutrophils based on NETs formation. The y-axis represents cellular counts of anti-citrullinated histone H3. **(C)** RNA-seq transcript expression of NETs forming neutrophils and non-NETs forming neutrophils from control animals (NF-C & NNF-C) and T2D animals (NF-T & NNF-T) represented as volcano plots with ln-transformed gene expression (x-axis) and the FDR-adjusted negative log-transformed p-value (y-axis). **(D)** Manhattan plot representing the change in gene expression in NETs forming neutrophils compared to non-NETs forming neutrophils in control and T2D. The y-axis represents the p-value, and the x-axis represents the chromosomes

We next sought to determine transcriptional basis for constitutive NETosis in T2D. Hence, we established a protocol to separate NETs forming (NF) from non-NETs forming neutrophils (NNF) derived from bone marrow in response to LPS in both control and T2D models. These subsets were separated based on cells expressing citrullinated histones using a magnetic bead-based selection strategy (Fig. 3A) and confirmed using flow cytometry (Fig. 3B). The RNA obtained from these two sub-populations was further processed for RNA sequencing (Fig. S10A).

**Fig. 4:**
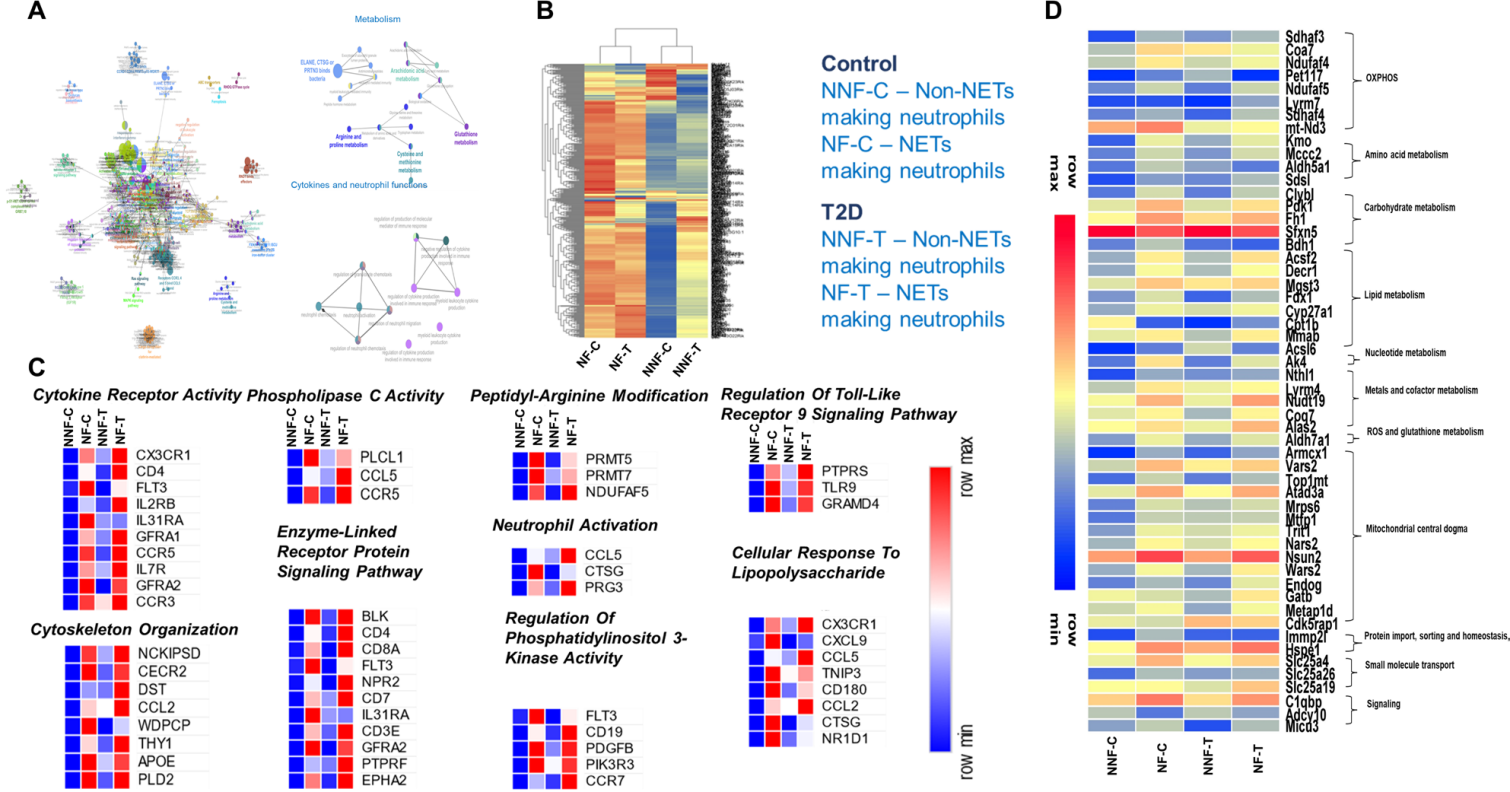
Bioinformatics analysis using differentially expressed genes between the subsets **(A)** Gene data set obtained was used for GO gene enrichment profiling using Cytoscape on the basis of ClueGO. The network obtained shows the enriched biological process, molecular functions and pathways connected by nodes. The size of the nodes determines the number of gene hits for the following pathway (Fig. SF11A). Edges connecting these terms are based on shared genes between the GO (kappa score = 0.5). Statistical significance was performed using the Enrichment/Depletion two-sided hypergeometric test, and the p-value was corrected using the Bonferroni step-down method. **(B)** Hierarchical clustered heat map representing distinct transcriptomic profiles of NF and NNF in control and T2D. Log CPM values were used to plot the heatmap, and colour change from blue to red indicates the upregulation of genes. **(C)** Heat maps representing differentially expressed genes enriched in biological processes and pathways contributing to NETs formation are provided. Colour intensity from blue to red shows the upregulation of the gene based on logCPM values. **(D)** Mitochondrial gene clustered to the respective function is plotted as a heatmap to compare the expression of the gene dataset between NF and NNF neutrophil population of control and T2D animals. Colour intensity from blue to red represents an increase in the logCPM value of the genes

Further, differential gene expression analysis was performed to identify pathways that are significantly modulated in neutrophil subpopulations with or without the ability to form NETs in control and T2D animals. A Manhattan plot revealed the dispersion of upregulated and downregulated genes across chromosomes between NF and NNF in both groups (Fig. 3D). Hierarchical clustering represents a distinct transcriptome profile of NF and NNF in the control and T2D (Fig. 4B). Data from control animals indicated that a total of 569 genes were upregulated and 107 were downregulated in NETs forming neutrophils compared to the cells with the inability to form NETs (Fig. 3C). In T2D animals, NETs forming neutrophils exhibited upregulation in the expression of 329 genes and downregulation of 115 genes compared to non-NETs forming neutrophils (Fig. 3C).

We next performed pathway and gene ontology (GO) enrichment analysis considering the significantly modulated genes (-1.5 > logFC > 1.5, p-value < 0.05) in neutrophils with the ability to form NETs using ENRICHR and to identify key pathways, biological processes, and functions. NETs making neutrophils showed high expression of genes enriched for pathways including RND1 GTPase cycle (*p* = 0.0001651), classical antibody-mediated complement activation (*p* = 0.0004297), chemokine receptors bind chemokines (*p* = 0.001008), *RUNX3* regulates WNT signalling (*p* = 0.001153); Biological process - Histone methylation (*p* = 0.00007289), cellular defense response (*p* = 0.0003890), peptidyl-arginine modification (*p* = 0.0004297); Molecular functions - cytokine receptor activity (*p* = 0.00005670), chemokine receptor activity (*p* = 0.001788), G protein-coupled chemoattractant receptor activity (*p* = 0.005294). Downregulated genes involved in the molecular functions were calcium-dependent phospholipid binding (*p* = 0.02622), arginine binding (*p* = 0.03686), and sodium channel inhibitor activity (*p* = 0.03686). Corresponding genes of these pathways and functions are summarized in Supplementary Table 1.

NETs forming neutrophils in T2D animals showed genes enriched in the pathways including RUNX1 regulates transcription of genes involved in BCR signalling (*p* = 0.003874), regulation of complement cascade (*p* = 0.006302); Biological process - enzyme-linked receptor protein signaling pathway (*p* = 1.359e-7), positive regulation of kinase activity (*p* = 0.00005518), calcium ion transmembrane import into cytosol (*p* = 0.0004355); Molecular functions - voltage-gated calcium channel activity (*p* = 0.0001152), protein tyrosine kinase activity (*p* = 0.0002301), cytokine receptor activity (*p* = 0.0002529). Downregulated genes were involved in the pathways which included extracellular matrix organization (*p* = 0.02650), mitochondrial uncoupling (*p* − 0.03401); Biological functions - positive regulation of cell communication (*p* − 0.01284), positive regulation of membrane potential (*p* − 0.03957); Molecular functions - arachidonate-CoA ligase activity (*p* = 0.02842) and voltage-gated calcium channel activity (*p* = 0.02995).

Subsequently, we looked for differentially expressed genes that activate unique pathways responsible for constitutive NETosis in T2D animals. The upregulated genes associated with the biological process included calcium ion import across the plasma membrane (*p* = 0.0002441), extracellular structure organization (*p* = 0.0002988), calcium ion transmembrane import into the cytosol (*p* = 0.0006389), positive regulation of microtubule nucleation (*p* = 0.001399). Molecular functions influenced by these genes involved high voltage-gated calcium channel activity (*p* = 0.000001106) and voltage-gated calcium channel activity (*p* = 0.00004291).

Further as we mined deeper into the GO analysis using ClueGO to identify functionally organized pathway networks. The data revealed significant differences in the gene expression contributing to neutrophil functions corresponding to immune responses, granulopoiesis, neutrophil activation, neutrophil chemotaxis, cytokine production, cytokine mediated-signalling pathways, cytokine receptor activity, cell response to LPS, Phospholipase-C activity and cytoskeleton reorganization (Fig. 4C). Genes corresponding to the pathways showed significant modulations in NETs forming neutrophils in both control and T2D groups. Network analysis also indicated differential gene expression in metabolic processes such as arachidonic acid metabolism, arginine and proline metabolism, cysteine and methionine metabolism and glutathione metabolism (Fig. 4A). The complete list of gene ontology terms and associated genes has been provided as a supplementary file (Supplementary Table 2). Further analysis also indicated significant changes in the genes controlling mitochondrial metabolism and functions which included oxidative phosphorylation (OXPHOS), mitochondrial central dogma and mitochondrial signalling (Fig. 4D). Collectively, these findings suggest significant alterations in gene-expression patterns within pathways linked to both metabolism and innate immune activation may be associated with changes in the immuno-metabolic axis.

### Alteration in the immuno-metabolic axis in T2D are associated with reduced response of neutrophils during sepsis

**Fig. 5:**
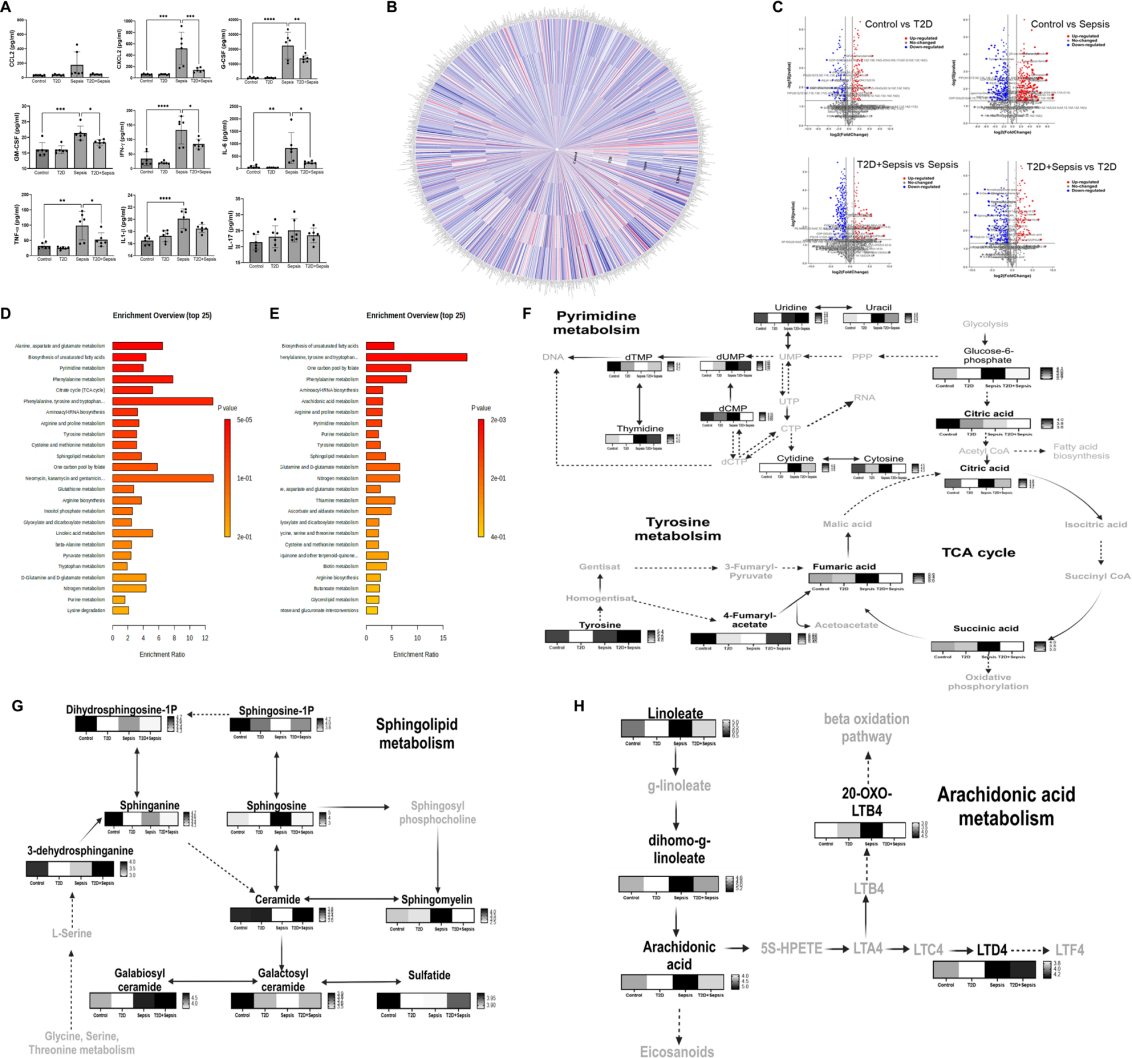
T2D animals show distinct systemic metabolic patterns during steady state and sepsis. Plasma from Control (*n* = 6), T2D (*n* = 6), Sepsis (*n* = 6) and T2D + sepsis (*n* = 6) were processed for measurement of inflammatory mediators responsible for granulopoiesis and untargeted metabolomics. **A**. Plasma levels of indicated cytokines were measured by multiplex ELISA and data is represented as pg/mL. Statistical significance is represented as asterisk * = *p* < 0.01, ** = *p* < 0.001, *** = *p* < 0.0001. **(B)** Circular heatmap plotted using whole spectral features represents plasma metabolome of above-mentioned study groups. **(C)** Volcano plots constructed based on Log2 (abundance ratio) and antilog P value shows differential metabolites among various comparisons. **D**&**E**. Enrichment Analysis showing the highly altered metabolic pathways in sepsis with respective to control and T2D + sepsis with respective to sepsis are provided. Heatmaps representing differentially expressing metabolic intermediates of TCA cycle (**F**), pyrimidine metabolism (**F**), tyrosine metabolism (**F**), sphingolipid metabolism (**G**) and arachidonic acid metabolism (**H**) are provided. Colour intensity from black to white represent decrease in the log abundance value

To understand key immuno-metabolic processes contributing to impeded neutrophil responses in T2D during infection, we measured inflammatory mediators regulating steady state and emergency granulopoiesis and simultaneously performed untargeted metabolomics in the plasma of T2D mouse models with or without inducing sepsis. Chemokines such as CCL2 and CXCL2 regulate neutrophil expansion in the bone marrow. Sepsis induction led to a nearly four-fold increase in CXCL2 levels (517.19 ± 181.98 pg/mL; *p* < 0.0001) and interestingly, CXCL2 levels did not alter in T2D animals upon the induction of sepsis. CCL2 levels also followed similar patterns as those of CXCL2, where T2D animals did not show elevation upon sepsis induction. Granulopoiesis stimulating cytokines G-CSF (22419 ± 9049.06 pg/mL vs. control 633.1 ± 525.36 pg/mL) and GM-CSF (21.38 ± 2.2 pg/mL vs. control 16.11 ± 2.2 pg/mL) were significantly increased in the sepsis and in T2D animals, induction of sepsis failed to produce these cytokines. Pro-inflammatory cytokines regulating emergency granulopoiesis such as IFN-γ (132.9 ± 48.08 pg/mL) vs. control (34.37 ± 23.38 pg/mL), IL-6, (825.3 ± 622.7 pg/mL) vs. control (71.13 ± 52.42 pg/mL); TNF-α (98.16 ± 46.6 pg/mL) vs. control (31.53 ± 9.6 pg/mL) and IL-1β (20.11 ± 1.6 pg/mL) vs. control (16.49 ± 0.6 pg/mL) were significantly increased upon induction of sepsis. However, T2D animals upon sepsis induction, did not show enhanced expression of these cytokines. IL-17 has been demonstrated to regulate granulopoiesis by stimulating GM-CSF and controlling the mobilization of neutrophils from bone marrow. The sepsis group showed an increasing trend in IL-17 levels and followed a similar pattern like other cytokines, however insignificant (Fig. 5A). These findings revealed that T2D animals show reduced levels of granulopoiesis-associated inflammatory mediators in response to sepsis.

Next, to determine the metabolic basis of T2D animals showing reduced response to sepsis, we carried out plasma metabolomics analysis in all four groups of animal models: (a) healthy (b) sepsis (c) T2D (d) T2D + sepsis. LC-MS analysis revealed 10,193 spectral features extracted from the raw data acquired in positive ionization mode. Principal component analysis (PCA) revealed significant stratification of the experimental groups, suggesting that each group contained distinct metabolic profiles (Fig. S12A). The control, T2D and sepsis group formed distinct clusters and the T2D with sepsis animals were aligned in the quadrant with the T2D animals. After annotating small molecules based on mass/charge within a tolerance of 15 ppm and excluding xenobiotic compounds, 1271 endogenous metabolites were considered for further analysis. These comprised 974 lipids, 143 amino acids and their derivatives, 17 carbohydrates, 46 intermediates of nucleotide metabolism and 74 other compounds. Circular heatmap representation (Fig. 5B) revealed differential levels of metabolites among groups. Volcano plots generated using univariate analysis indicated significant alterations among groups (Fig. 5C). In order to validate the annotations, pooled samples were subjected to ms/ms analysis to obtain the fragmentation patterns (Supplementary Table 3).

Further, pathway enrichment analysis of compounds significantly modulated in sepsis in comparison with healthy animals showed substantial modulation of alanine, aspartate and glutamate metabolism (*p* < 0.0001), pyrimidine metabolism (*p* < 0.0001), phenylalanine metabolism (*p* < 0.0001), biosynthesis of unsaturated fatty acid metabolism (*p* < 0.0001), sphingolipid metabolism (*p* < 0.001) and TCA cycle (*p* < 0.0001) (Fig. 5D). Sepsis with T2D background showed significant modulation of biosynthesis of unsaturated fatty acid metabolism (*p* < 0.0001), phenylalanine, tyrosine and tryptophan metabolism (*p* < 0.0001), arachidonic acid metabolism (*p* < 0.0001) and sphingolipid metabolism (*p* < 0.01) in comparison with T2D animals (Fig. 5E). We observed that lipid metabolism, which is involved in inflammatory responses, was significantly elevated in the sepsis model and negatively regulated in T2D with sepsis.

Sphingolipids are an extensively diverse group of lipids that regulate cell structure, growth, survival, and immune cell trafficking. Intensities obtained for sphingolipid pathway intermediates show altered distribution in sepsis with or without T2D. Sphingosine was found to be significantly increased in sepsis (*p* = 0.0001) and decreased in T2D with sepsis animals (*p* < 0.0001). Other intermediates such as sphingomyelin (d18:0/14:0) and galabiosylceramide were significantly increased in sepsis (*p* < 0.0001). During sepsis in a T2D background sphingomyelin (d18:0/14:0) was significantly decreased (*p* < 0.0001) in comparison to sepsis (Fig. 5G).

Anti-inflammatory lipid pathways such as the arachidonic acid metabolic pathway, were also modulated during sepsis and sepsis with T2D background. Linoleic acid and arachidonic acid were increased in sepsis mice and were significantly decreased in T2D with sepsis mice (*p* < 0.0001). Leukotrienes are arachidonic acid metabolites derived from the action of 5-LO (5-lipoxygenase). 20-oxo-leukotreine B4, a metabolite of omega oxidation of Leukotriene B4 (LTB4), was significantly increased in sepsis (*p* < 0.0001) and T2D animals failed to accumulate these metabolites upon inducing sepsis (*p* < 0.0001) (Fig. 5H). Unsaturated fatty acids such as oleic acid (*p* < 0.0001), α-linoleic acid (*p* < 0.0001), and dihomo-γ-linoleate (*p* < 0.001) were significantly reduced in T2D with sepsis models compared to sepsis alone (Fig. 5H). TCA cycle intermediates also showed significant perturbations among sepsis and T2D + sepsis animals. Succinic acid (*p* < 0.0001), citric acid and fumaric acid (*p* < 0.0001) were significantly increased in sepsis. However, T2D animals failed to show an increase in TCA cycle intermediates upon inducing sepsis (*p* < 0.0001) (Fig. 5F). In summary, we observed significant differences in levels of metabolic intermediates when sepsis was induced in either healthy and T2D background.

### Correlation patterns of inflammatory mediators regulating granulopoiesis and metabolites are distinct in animals induced with sepsis in either healthy or T2D background

**Fig. 6:**
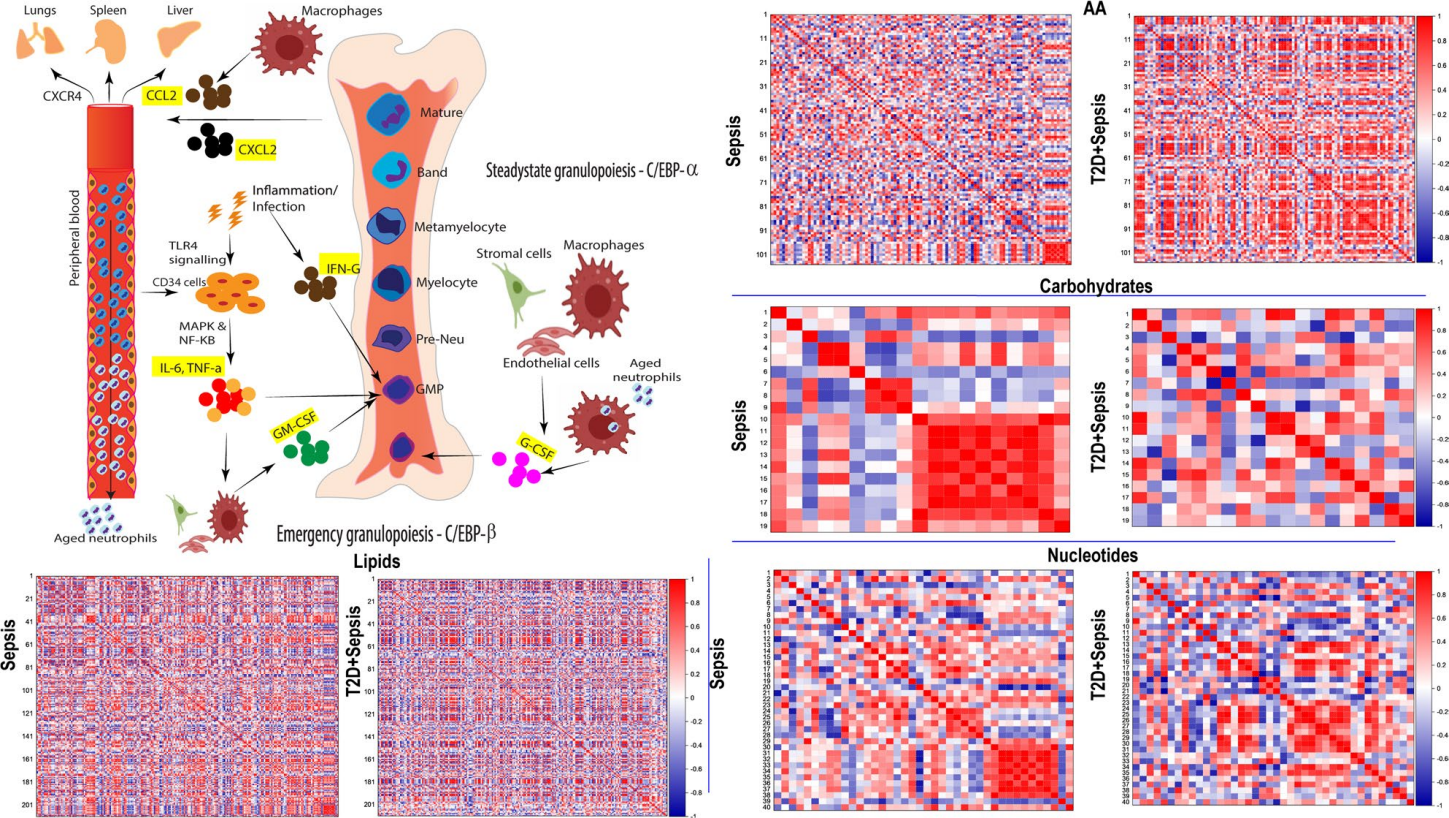
Sepsis induced in T2D animals show perturbed immuno-metabolic patterns. **A**. Schematic representation showing role of multiple cytokines in life cycle of neutrophils. **B**. Pearson’s correlation analysis was performed using abundance of plasma metabolites and cytokines from sepsis and T2D + sepsis animals. Separate correlation plots for lipids, amino acids, carbohydrates and nucleotides are shown. The correlation plots provided contains data for only metabolites which are showing correlation coefficient of greater than ≥ + 0.5 and ≤-0.5 for amino acids, nucleotides, carbohydrates and ≥ + 0.75 and ≤-0.75 correlation coefficient for lipids showing significant correlation to atleast one of the inflammatory mediators. Colour intensity from white to red indicates increase in the association whereas white to blue indicates negative association between metabolites and cytokines. The complete list of metabolite-cytokine correlation and selected correlations for the plot are given as excel sheet in supplementary (Supplementary Tables 4, 5, 6, 7). Statistical significance was adjusted using Benjamini–Hochberg false discovery rate (FDR) procedure

Accumulating evidence shows that metabolic and immune response pathways are regulated interdependently and modulate the course and severity of inflammation. Pearson’s correlation analysis revealed a significant association between metabolic and inflammatory mediators regulating granulopoiesis. Amino acids and derivatives showed 96 correlations with cytokines in sepsis and 22 associations in T2D + sepsis were dysregulated (Fig. 6). Valyl-hydroxy-proline, methionyl-methionine, alanyl-proline, pyroglutamic acid were negatively correlated with all cytokines in sepsis and these correlations turned positive in T2D + sepsis animals. N-lactoyl-methionine, lysyl glycine, N-formyl-L-glutamic acid were positively correlated in sepsis, whereas these correlations were negatively associated in T2D + sepsis. IL-6, TNF-α, IL-1β, and INF-γ, which regulate emergency granulopoiesis, when correlated with metabolites showed negative or no association in sepsis with T2D background. L-tryptophan, N-acetyl-L-phenylalanine, L-proline, glyceric acid, L-homocysteine and phenylalanine showed positive correlation with IL-6 in sepsis mice and these correlations were reversed in T2D + sepsis. Upon inducing sepsis, correlations with glutaminoyl methionine, L-tyrosine, L-methionine, fumaric acid and L-asparagine were intensified with IL-6, TNF-α, IL-1β, INF-γ and these associations were either lost or negatively correlated in T2D + sepsis. This indicates that such a perturbation in immunometabolism may contribute to the reduced ability of T2D animals to mount an immune response against sepsis.

Lipid metabolism significantly regulates the course and severity of infection and sterile inflammation. Similar to the correlation patterns of amino acid derivatives - cytokines, lipids-cytokine correlations were also perturbed in T2D animals upon inducing sepsis. Cardiolipins (CL) are mitochondrial phospholipids that modulate immune functions during inflammation. Our data identified 60 cardiolipins, and among them, 10 were negatively correlated with all the cytokines and 5 were positively correlated with sepsis. However, these correlations were lost in T2D + sepsis animals, where 12 and 3 cardiolipins were positively and negatively correlated, respectively. Cytidine diphosphate - diacylglycerol (CDP-DG) is a primary component to produce cardiolipin, phosphatidylinositol and phosphatidylglycerol. We identified 79 CDP-DG’s and 29 correlations were positively associated with cytokines in sepsis and all CDP-DG molecules were either negatively correlated or no association was found in T2D + sepsis animals (Fig. S13A). Ceramides are central molecules in the biosynthesis and metabolism of sphingolipids. Our data showed enrichment of 42 ceramides. Other than glucocerebroside, 8 ceramides were negatively correlated in sepsis and differentially modulated in T2D + sepsis. IFN-γ and IL-6 positively correlated with all the ceramides in the T2D + sepsis condition. Several studies have reported anti-inflammatory properties of ganglioside, specifically GM3 (Fig. S14A). A total of 17 gangliosides were identified, and three each gangliosides’ species were positively and negatively correlated with various cytokines in the sepsis model. IL-6 was found with no correlation with gangliosides. On the other hand, TNF-α, GM-CSF, IL-1β, and CXCL2 were positively correlated with a minimum of 3 gangliosides and negatively correlated with the other 3. Sphingosine, which is a key intermediate in the sphingosine metabolic pathway, was positively correlated with all the cytokines. During sepsis in a T2D background, sphingosine was found negatively correlated or lost the correlation. Association between carbohydrate metabolites and cytokines also showed modifications from sepsis to T2D with sepsis. Beta-D-fructose 2,6 bisphosphate and UDP-4-hydro-6-deoxy-D-glucose were positively correlated with IL-6 in sepsis and negatively correlated in T2D with sepsis. Intermediates of nucleotide metabolism including 3’, 5’-Didioxythymidine, 2’-o-methylcytidine, cytidine, N2-methylguanine, 2’-C-Methylguanosine, 3’-deoxythymidine-5’-monophosphate and xanthine were positively correlated with all the cytokines in sepsis condition. These metabolites either lost their correlation or negatively correlated in the T2D + Sepsis condition. 2’, 3’-deoxythymidine triphosphate, dCMP, and guanosine triphosphate adenosine were negatively correlated with cytokines in sepsis and positively correlated with T2D with the infection model (Fig. 6). These findings demonstrate dysregulated immuno-metabolism in T2D + sepsis animals may be associated with reduced responsiveness to infections.

### Molecular dynamics simulations of the mitochondrial membrane in disease conditions

**Fig. 7.**
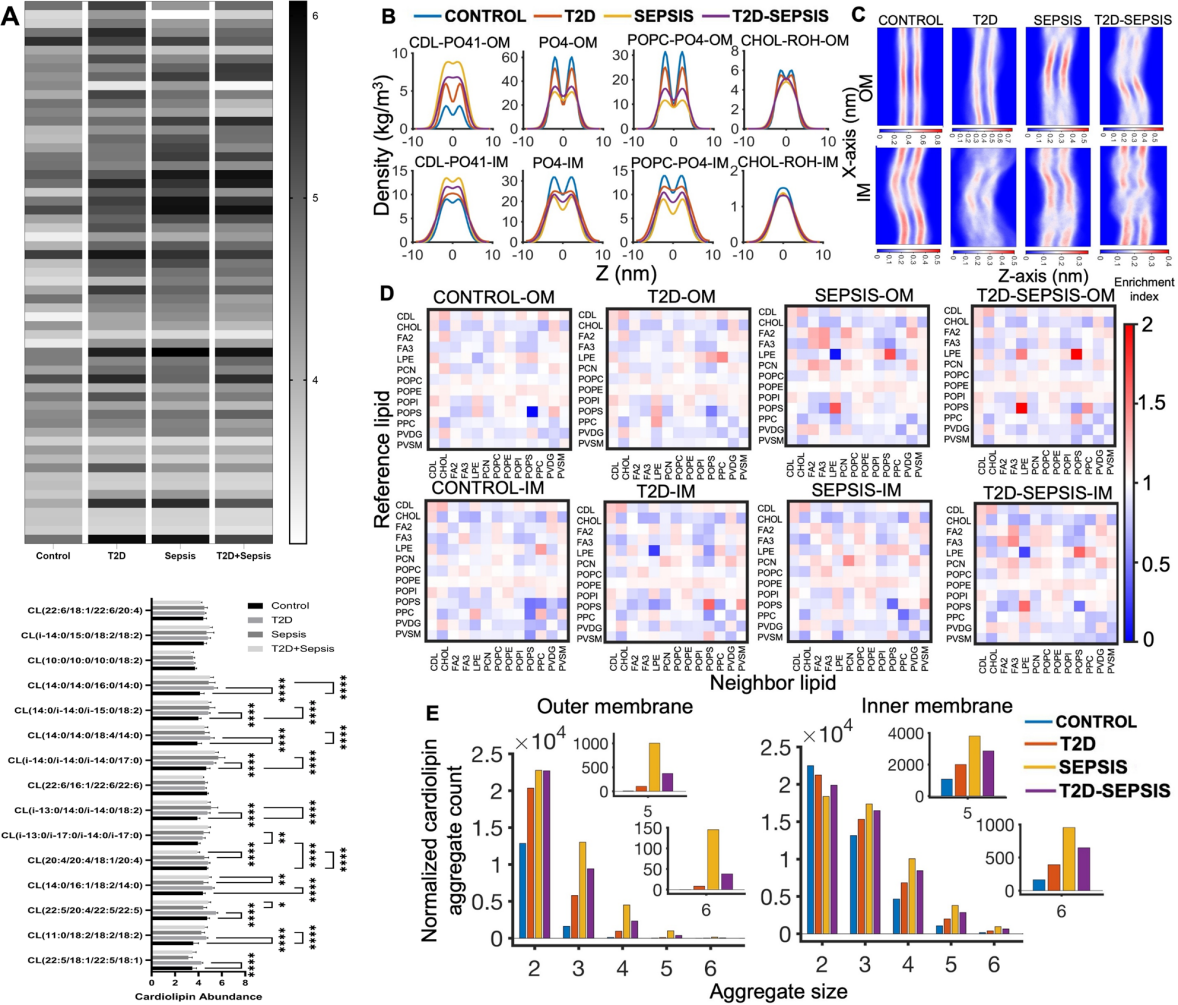
Membrane structure of mitochondria is altered in T2D **A**) Heatmap representing differentially expressing cardiolipin species is provided. Colour intensity from black to white represent decrease in the log abundance value. Bar graph represents significantly correlated cardiolipins ( ≥ + 0.75 and ≤-0.75 correlation coefficient) to at least one of the cytokines. Statistical significance was obtained by performing two-way ANOVA followed by Tukey’s multiple comparison test to control the family-wise error rate. Adjusted p-values are represented as asterisk. (* = *p* < 0.01, ** = *p* < 0.001, *** = *p* < 0.0001). (**B**) Density profile of membrane lipid components along the membrane normal direction, averaged over the concatenated trajectory of 6 µs combining the three replicas of each system. The color codes that define different diseased conditions are: control: Blue, T2D: Orange, sepsis: Yellow, T2D-sepsis: Violet. **C**) Number density profile of phosphate beads (PO4) in the membrane bilayers averaged across 2 µs of each MD simulation along the y-axis as determined using Gromacs tools. In the plot, regions of higher density are shown in red, and areas of lower density in blue. **D**) Lipid enrichment contact-map of lipids in the membrane bilayer using a cut-off of 1.2 nm. The analysis is carried out on the entire 6 µs trajectory of individual systems (by concatenating the simulated replicas). Higher enrichment values (more than 1) indicate areas where the specific neighbor lipid type is more concentrated around the reference lipid. **E**) Cardiolipin near-neighbor aggregate count was obtained from the concatenated simulation trajectory of 6 µs using a cut-off of 1.2 nm. The normalized cardiolipin aggregate count refers to the cardiolipin aggregate count divided by the number of cardiolipins. The color codes that define different diseased conditions are: control: Blue, T2D: Orange, sepsis: Yellow, T2D-sepsis: Violet

Mitochondrial membrane lipids, cardiolipins drive inflammation by targeting TLR4 receptors. Our data annotated 61 cardiolipins (Fig. 7A), and 15 of them were positively correlated with inflammatory mediators that regulate granulopoiesis. Interestingly, these cardiolipin species showed differential abundance between sepsis and T2D + sepsis groups (Fig. 7A). Mitochondrial functions are modulated by constant fusion and fission, which regulate their dynamics, a critical process of mitochondrial bioenergetics. Cardiolipins are an integral part of the mitochondrial membranes and drive mitochondrial dynamics [31]. We employed multiple coarse-grained molecular dynamics (CG-MD) simulations on the timescale of microseconds (µs) to investigate the impact of altered cardiolipins (CDLs) composition in different diseased states on the structure and organizational dynamics of both the outer and inner mitochondrial membranes. We considered control, T2D, sepsis, and T2D-sepsis conditions.

The membrane composition of control system was based on a previous study [26]. The membrane bilayers in different diseased conditions were modelled based on the percentage fold change values of lipid components (Cardiolipin and 1-palmitoyl-2-oleoyl-sn-glycero-3-phosphocholine, POPC) with respect to the control, as obtained from the rat metabolome data. Details of the lipid bilayer compositions are shown in supplementary Table 8. We observed that changes in the cardiolipin concentration significantly impacted the bilayer properties (Fig. S15A). In particular, aberrations were observed in the mitochondrial inner membrane in T2D conditions. In sepsis conditions, the area per lipid increased significantly in response to high cardiolipins, suggesting a structural alteration in the mitochondrial membrane in response to sepsis. Again, the response is not pronounced in the T2D-sepsis condition, compared to the sepsis-only condition. The inner membrane was particularly perturbed, evident from the violin plot distribution in the case of the T2D-sepsis model. Furthermore, the lipid density profiles exhibited notable variations depending on the disease condition. As shown in Fig. 7B, the density distribution of lipid components across the bilayer was relatively uniform for the control, suggesting a less perturbed membrane. However, the lipid density profile was significantly perturbed in the cases of T2D, sepsis, and T2D + sepsis conditions. Compared to the control condition, there was a significant density of cardiolipin (PO41 bead) and phosphates (PO4) in the middle of the bilayer in sepsis and T2D-sepsis conditions. Figure 7C shows the number density profile of phosphate beads (PO4) in the bilayer averaged over 2 µs of simulation trajectory for each system. In control membranes, the distribution of PO4 beads was uniform in both outer and inner mitochondrial membranes. The inner membrane is more flexible, as it is known to form cristae. When sepsis conditions were modelled, both inner and outer mitochondrial membranes became more flexible but with clearly defined lipid densities. This suggests increased mitochondrial dynamics without bilayer structure deformation. In the case of T2D, the lipid densities were deformed, particularly in the inner mitochondrial membrane, suggesting mitochondrial aberration. In the case of the T2D-sepsis condition, the inner membrane was clearly disrupted, and even fragmentations were observed.

We calculated the lipid enrichment in each bilayer to determine the extent of heterogeneity in the lipid distribution across the bilayer (Fig. 7D). We used lipid enrichment heatmaps, averaged over the entire 6 µs simulation trajectory, with a cut-off distance of 1.2 nm. Higher enrichment indicates a better probability of spatially finding a lipid around a lipid of interest. Our data demonstrates that although we primarily changed the cardiolipin composition to mimic different diseased conditions, overall spatiotemporal organization changed in different disease conditions, which might be the origin of drastic changes in mitochondrial dynamics in response to sepsis in different backgrounds (Control/T2D). Enrichments of cardiolipins around themselves were observed in Fig. 7D, suggesting the formation of cardiolipin aggregates. This enrichment was also observed with cholesterol, which tends to accumulate near cardiolipins. Apart from cholesterol, lipids such as POPI, PPE, and LPE were also found to be enriched around cardiolipins. To quantify the extent of aggregation within the bilayers, we calculated the sizes of cardiolipin aggregates. In the control condition, the cardiolipin aggregates size of 6 was minimal in number (Fig. 7E). Specifically, the number of cardiolipin clusters larger than size 4 was not significant in the outer and inner mitochondrial membranes for control (Fig. 7E). Notably, we observed a substantial increase in higher-order cardiolipin aggregates in the diseased conditions (T2D, sepsis, and T2D-sepsis) (Fig. 7E). In sepsis, we observed the highest number of higher-order cardiolipin-aggregates (Fig. 7E). This aggregate might be pivotal in controlling mitochondrial dynamics in response to sepsis. However, the extent of the cardiolipin aggregation was less in T2D-sepsis conditions, indicating the different spatiotemporal organization, which may lead to altered mitochondrial dynamics when sepsis is introduced in T2D subjects. Our simulation data show altered dynamics and organization of mitochondrial membranes, particularly the inner membrane, in T2D conditions that may lead to mitochondrial aberration.

### T2D state leads to the accumulation of defective mitochondria in neutrophils

**Fig. 8:**
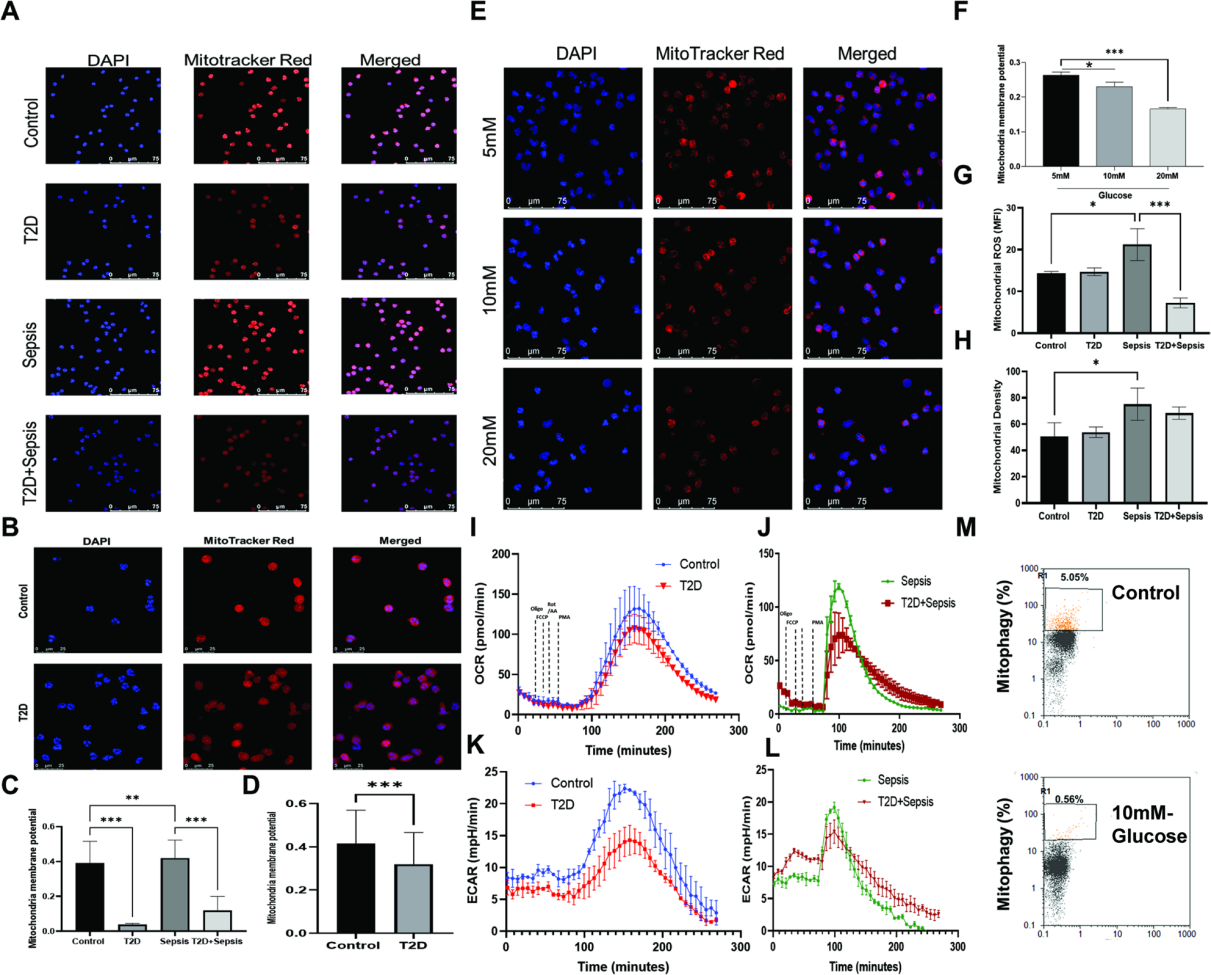
Mitochondrial functions are constitutively compromised in T2D state and show reduced response upon induction of sepsis. Neutrophils isolated from peripheral blood of mouse models (*n* = 12; 3 in each group) (**A**) and humans (*n* = 4, control = 2; T2D = 2) (**B**) were stained for nucleus (DAPI) and mitochondrial membrane potential (MitoTracker Red CMXRos) and processed for confocal imaging. Representative microscopy images are provided; Scale = 75 μm. **C.** Graphical representation of the cumulative intensity readings of MMP collected from neutrophils; control (*n* = 136), T2D (*n* = 125), sepsis (*n* = 180), T2D + sepsis (*n* = 114) is provided. **D.** Bar graph represents the cumulative intensity readings of MMP from each neutrophil; healthy (*n* = 77) and T2D (*n* = 139). **E.** Neutrophils from healthy individuals (*n* = 2) were treated with indicated concentrations of glucose for 2 h and stained for MMP and nucleus. Representative confocal images are provided. Scale = 75 μm. **F**. Data is represented as arbitrary units obtained from neutrophils treated with 5mM (*n* = 243), 10mM (*n* = 141) and 20mM (*n* = 305) concentration of glucose. Neutrophils isolated from different animal groups were subjected to estimation of ROS (**G**) and mitochondrial density (**H**) by flowcytometry using mitoSOX and nonyl acridine orange dyes respectively. Data is represented as mean fluorescence intensity. **I,J,K,L**. Extracellular flux analysis was performed in neutrophils isolated from healthy and T2D subjects. Approximately 40,000 cells/well were seeded in poly-Lysine coated plates and cultured in RPMI without serum followed by recording of oxygen consumption. Data represents area under the curve of total oxygen consumption of healthy (*n* = 2), T2D (*n* = 2), sepsis (*n* = 2) and T2D + sepsis (*n* = 2) subjects. **M.** Dot plot represents neutrophils isolated from healthy individuals (*n* = 2) which were treated with indicated concentrations of glucose for 2 h and stained for mitophagy

As both RNAseq and metabolomics data revealed enrichment of intermediates associated with TCA cycle, cardiolipins and OXPHOS pathways, we asked whether mitochondrial functions are perturbed in T2D neutrophils during infections. Confocal imaging using mitotracker red CMXRos revealed a reduced mitochondrial membrane potential (MMP) in neutrophils isolated from T2D animals (0.037 *±* 0.007 AU) compared with the control (0.39 *±* 0.12 AU) (Fig. 8A & C). Further, we observed a significant increase in the MMP upon the induction of sepsis (0.419 *±* 0.103 AU) and interestingly, when sepsis was induced in T2D animals, neutrophils failed to further enhance MMP (0.119 *±* 0.07 AU) (Fig. 8A & C). Consistent with our findings in the animal models, human neutrophils isolated from T2D subjects (0.319 *±* 0.146 AU) also displayed a defective MMP against healthy neutrophils (0.415 *±* 0.153 AU) (Fig. 8B & D). Further, we observed a gradual decrease in the mitochondrial activity in response to hyperglycaemia (5.5mM vs. 10mM, p value < 0.05; 5.5mM vs. 20mM, p value < 0.001) (Fig. 8E & F). These results suggest hyperglycaemia is associated with mitochondrial dysfunction in neutrophils. Subsequently, we quantified constitutive levels of mitochondrial reactive oxygen species (mROS) in the neutrophils. Induction of sepsis led to a significant increase in mROS in neutrophils (21.20 *±* 3.11 MFI vs. Control – 14.32 ± 0.33 MFI) (Fig. 8G). In contrast, a substantial reduction was observed in the T2D + sepsis model (7.26 *±* 0.96 MFI vs. sepsis 21.20 *±* 3.11 MFI) (Fig. 8G). Sepsis induction increased mitochondrial mass in both diabetic and non-diabetic models. However, the elevation of mitochondrial mass was more prominent when sepsis was induced in control animals (75.17 *±* 10.04 vs. Control – 50.75 *±* 8.41 MFI). (Fig. 8H). This suggests that mitochondrial density in control and T2D during sepsis remains unchanged; however, mitochondria in T2D neutrophils shows reduced functional capacity.

NETosis is a redox sensitive process and utilize ROS derived from either NADPH oxidase or mitochondria, based on stimulus. Neutrophils robustly consume superoxide anions derived from NADPH oxidase upon activation through a process referred to as oxidative burst. As mitochondrial function was compromised in T2D neutrophils, we performed extracellular flux analysis to assess oxidative burst in response to PMA upon inhibiting the electron transport chain. We recruited four groups of human subjects: (a) healthy individuals, (b) T2D-uninfected (c) sepsis and (d) T2D with sepsis. In response to PMA, healthy neutrophils (*n* = 2) (Fig. SF16) showed an increase in OCR levels which reached the highest activity at average of 180 min. However, T2D neutrophils displayed reduced levels of OCR when stimulated with PMA compared to control (Fig. 8I). Further, we compared OCR levels in neutrophils isolated from diabetic and non-diabetic individuals with sepsis. We observed neutrophils from non-diabetic individuals upon sepsis showed an elevation in OCR levels in response to PMA. The highest OCR levels of neutrophils isolated from non-diabetic individuals upon sepsis reached 109.52 *±* 40.8 pmol and T2D subjects with sepsis showed reduced OCR levels of 62.41 *±* 19.4 pmol (Fig. 8J). Further we also observed lower levels of extracellular acidification rate (ECAR) in T2D subjects with or without sepsis (Fig. 8K & L). This indicates the inherent dysregulation of the energy system in T2D neutrophils and were unable to respond during infections.

As we observed a decrease in mitochondrial membrane potential and no change in the mass, we suspected that impaired mitophagy may be involved in the accumulation of dysfunctional mitochondria. Our preliminary results suggests that constitutively 5% of neutrophils were positive for mitophagy, and further treatment with high glucose (10mM) showed a significant reduction in mitophagy levels (Fig. 8M). Taken together, these results suggested that hyperglycaemia is associated with reduced mitochondrial function (MMP and mROS), impaired mitophagy, and the accumulation of dysfunctional mitochondria, indicating that T2D conditions deteriorate mitochondrial bioenergetics required for efficient effector functions of neutrophils.

### Arachidonic acid induces mROS levels and restores NETs formation in T2D subjects

**Fig. 9:**
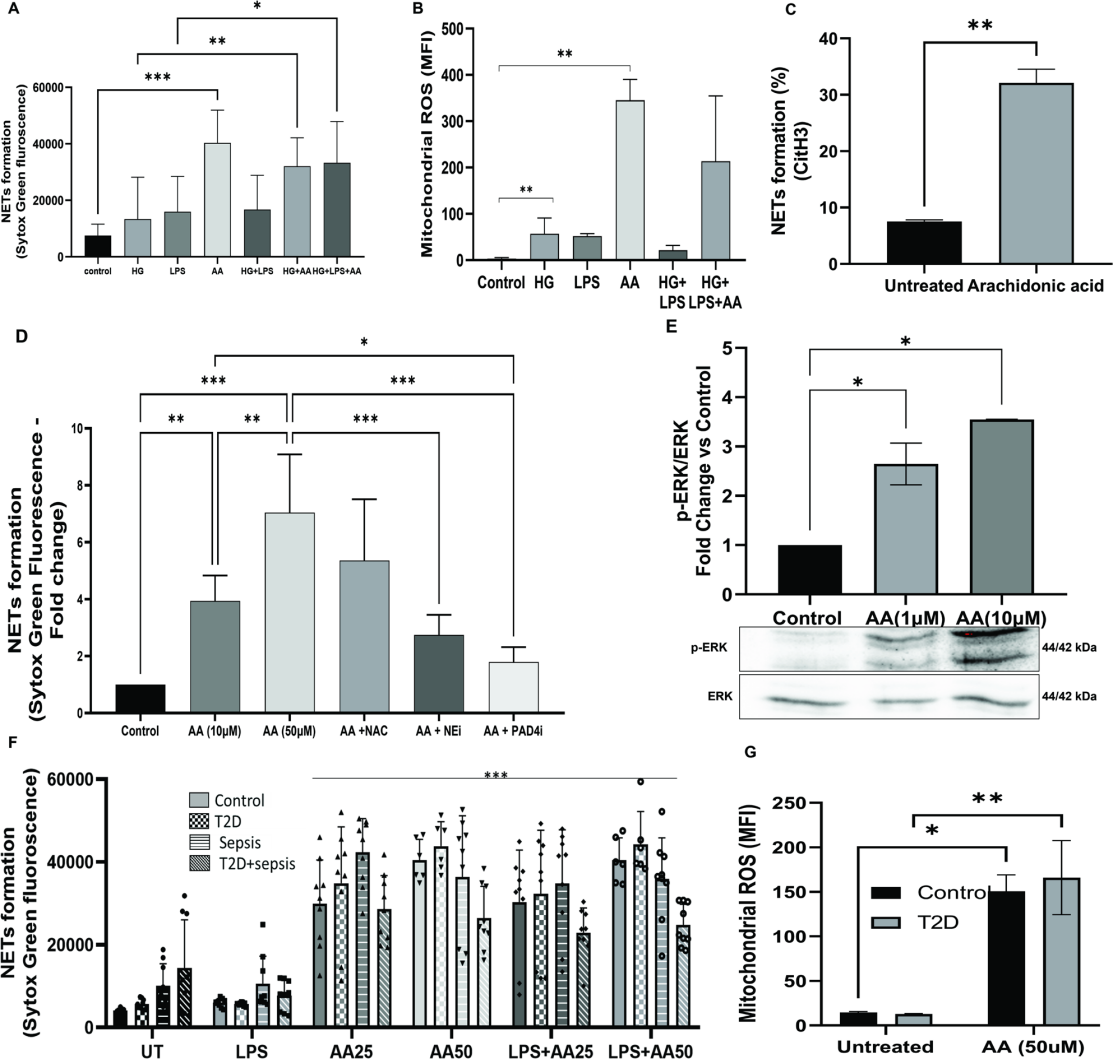
Neutrophils isolated from healthy individuals were seeded (50,000 cells/well) in the 96 well plate and incubated with arachidonic acid (AA), high glucose (HG), LPS and NAC for fluorimetry analysis and stained with mitoSOX for flowcytometry. **A.** Fluorescent intensity of the sytox green staining obtained from the neutrophils treated with high glucose, LPS, AA were measured using fluorimetry and represented as bar graph. **B & G.** Mitochondrial ROS readings obtained from flowcytometry is represented as mean fluorescent intensity. **C**. Neutrophils isolated from control (*n* = 3) and T2D (*n* = 3) subjects were treated with AA, stained with anti-citrullinated histone H3 and followed by secondary antibody and processed for acquisition in flowcytometry. Data is represented as bar graph with abundance of neutrophils on the Y axis. **D.** Effect of NAC (10µM), Sivelestat – Elastase inhibitor (Nei) (5µM) and Cl-amidine - PAD4 inhibitor (PAD4i) (20µM) on sytox green fluorescent reading is recorded and represented as bar graph. **E.** Immunoblots of neutrophil lysates treated with AA 1 µM and 10 µM. Representative blots stained for phospho-ERK^1/2Thr202/Tyr204^ and total-ERK are provided along with representation of foldchange between control (*n* = 2) and AA (*n* = 2) treated groups as bar graphs. **F.** Neutrophils were isolated from healthy (*n* = 3), T2D (*n* = 3), sepsis (*n* = 3) and T2D + sepsis (*n* = 3) subjects were seeded (50000 cell/well) in the 96 well plate and incubated with arachidonic acid (AA) in presence or absence of LPS. Data is represented bar graph. Bars represent the mean *±* SD and, * = *p* < 0.01, ** = *p* < 0.001, *** = *p* < 0.0001

Subsequently, we examined whether reactivation of mitochondria can restore the effector functions of neutrophils. As earlier studies have shown that arachidonic acid (AA) induces mitochondrial ROS, we explored whether supplementing AA can increase the mitochondrial activity to enhance NETs formation in T2D individuals. Neutrophils in response to AA formed robust NETs and restored NETs formation in neutrophils pre-treated with high glucose in response to LPS (Fig. 9A). AA facilitated the formation of mROS (345.15 *±* 31.81 MFI) compared to untreated neutrophils (3.00 *±* 1.23 MFI) (Fig. 9B). We confirmed the NETs production by AA treated neutrophils with citH3 (Fig. 9C). Further, AA-induced NETs were found to be significantly reduced upon treatment with N-acetyl cysteine (NAC - scavenger of ROS), NE inhibitor and PAD4 inhibitor (Fig. 9D), showing that AA-induced NETs pathway was dependent on activating NE and PAD4 enzymes. Next, we validated these pathways with phosphorylation levels of ERK^1/2Thr202/Tyr20^. Neutrophils treated with different concentrations of AA (1 µM and 10 µM) induced 2.6- and 3.5-fold increase in ERK^1/2Thr202/Tyr20^ phosphorylation, respectively (Fig. 9E). These results indicate arachidonic acid induce NETs in ERK1/2/Elane/PAD4 dependent pathway.

On similar line as our animal models, human neutrophils from healthy, T2D, sepsis and T2D + sepsis subjects also displayed significant elevation in the level of NETs upon stimulation with AA. T2D and sepsis neutrophils showed 8-fold increase and T2D + sepsis neutrophils showed 3-fold increment in NETs formation (Fig. 9F). Furthermore, we also observed substantial elevation in the production of mROS upon stimulating healthy and T2D neutrophils with AA (Fig. 9G).

## Discussion

Experimental and clinical studies have shown significant adverse effects of NETs in the pathogenesis of diseases associated with sterile inflammation, as these structures induce thrombosis in stroke [32]; delayed wound healing [9] and reduced response to infections in T2D [11] carry cancer cells to distant organs to induce metastasis [33]; exacerbate neuro-inflammation in Alzheimer’s disease [34]. Hence, characterizing neutrophils with confined effector functions and further modulating specific populations, along with simultaneously restoring the ability to eliminate pathogens, may facilitate maintaining immune homeostasis in the aforementioned pathologies. Our previous findings showed that T2D subjects displayed constitutively increased NETs formation, which led to impeded response to infections. Hence, this instigated us to examine and characterize functional plasticity of neutrophils and how these subpopulations behave in rodent and clinical models of T2D with or without sepsis.

In the present study, we identified neutrophil subpopulations that are confined to either the formation of extracellular traps or phagocytosis in both blood and bone marrow. Transcriptome of bone marrow neutrophils with the ability to form NETs significantly differed from neutrophils lacking the potential to form NETs. In experimental and clinical models of T2D with or without sepsis, the proportions of these neutrophil subpopulations were significantly altered. These alterations were associated with perturbations in the immune-metabolic axis, including changes in inflammatory mediators regulating emergency granulopoiesis and shifts in metabolic intermediates of the arachidonic acid pathway, TCA cycle, and mitochondrial membrane dynamics. T2D neutrophils harboured dysfunctional mitochondria and showed reduced ROS levels, MMP and impeded mitophagy. Further, arachidonic acid supplementation to T2D neutrophils activated mitochondria, increasing ROS and enhancing NETs formation via ERK1/2/Pad4 signalling in response to LPS. This study identifies associations and potential mechanisms for impeded neutrophil response to infections in T2D and however, mechanistic studies and validation in larger human cohorts are warranted to comprehensively understand these pathological conditions.

Phagocytosis and NETosis are evolutionarily conserved and important effector functions of neutrophils. Although, cellular and signalling mechanisms regulating these processes have been extensively characterized, ‘why’, ‘when’ and ‘how’ neutrophils choose to form either extracellular traps or undergo phagocytosis to eliminate pathogens are poorly defined. However, studies have demonstrated functional heterogeneity of neutrophil and its relevance to choose either phagocytosis or NETosis. For example, Branzk et al., reported neutrophil sensing mechanism based on size of the pathogen. Large pathogens such as *Candida albicans* and *Mycobacterium bovis* aggregates are cleared by selectively producing NETs formation over phagocytosis [35]. In mouse models, aged neutrophils characterized by the elevated expression of CXCR4 and reduced levels of CD62L showed increase in the NETs formation [36].

Multiple studies have identified the heterogeneous nature of neutrophils based on buoyancy, cell surface markers, localization and maturity [15, 37]. Based on buoyancy, neutrophils are characterized as low-density neutrophils (LDNs) [38] and normal-density neutrophils. LDNs were first reported in patients with autoimmune diseases, and these subtypes show a proinflammatory phenotype [15, 39]. Single cell RNA sequencing revealed eight distinct subgroups of neutrophils in the bone marrow and peripheral blood with unique functions in healthy and subjects with burnt wounds [40]. Puga et al., demonstrated that B helper neutrophils residing in the perifollicular region of the spleen stimulated thymus independent antibody production and were transcriptionally different from circulating neutrophils [41]. Further authors showed that ICAM-1Hi, CD11bHi and CD62low neutrophils in the spleen were more prone to produce NETs [41]. Subpopulation of neutrophils expressing VEGFR1^+^, CxCR4^+^, and CD49d^+^ in hypoxic conditions promoted angiogenesis [42]. In gastric cancer cell lines, IFIT1 + tumor associated neutrophils stimulates the growth of cell-derived xenograft models [43]. Besides neutrophils restricted solely to either phagocytosis or NETs formation, our results also pointed out subsets performing both phagocytosis and NETosis, which may be due to vital NETs where neutrophils have been described earlier to form NETs and still have the ability to phagocytose pathogens [44]. In addition, we found neutrophils that did not perform either phagocytosis or form NETs and interestingly, this population showed significant plasticity upon inducing sepsis in healthy animals.

Growing evidences show compromised neutrophil functions in T2D characterized by highly abundant LDNs with pro-inflammatory properties [45], defective phagocytosis [46] and constitutive NETs formation [6, 11, 12]. Such neutrophil dysfunction in T2D leads to impeded responses to infections and is one of the major causes for morbidity and mortality in T2D [47–51]. The functional response of neutrophils significantly varied when sepsis was induced in control animals compared to that of the T2D background. For example, the proportions of neutrophils forming NETs and phagocytosis altered significantly between sepsis and T2D + sepsis. Sepsis induction in control animals resulted in a cytokine storm, which robustly elevated production of inflammatory mediators regulating granulopoiesis, however T2D animals showed severe impairment in synthesizing these cytokines in response to sepsis.

Several studies including our own, have demonstrated that chronic inflammation along with hyperglycaemia is associated with constitutively activated neutrophils and impaired response to infections [6, 52]. These observations suggest perturbed immuno-metabolic axis in T2D at both the systemic and cellular levels, significantly influencing neutrophil dysfunction during infections. Metabolic intermediates of sphingolipid and arachidonic acid pathways, along with the TCA cycle have been demonstrated to potentially regulate inflammation. These key metabolites showed differential responses when sepsis was induced in healthy mice compared to those of T2D animals. The sphingolipid pathway acts as a double edge sword during infections. Intermediates of sphingolipid pathways are required to drive the inflammation during the early phase of sepsis whereas, prolonged downregulation of sphingolipid 1 phosphatase (S1P) increases the inflammatory cytokines such as IL1-β leading to non-beneficial cytokine storm [53]. In response to TNF-α and (N-formyl-methionyl-leucyl-phenylalanine) (fMLP), S1P is secreted from inflamed epithelial cells and tissues to induce migration and the intracellular ceramide content in neutrophils [54, 55]. Ceramides are the central regulatory intermediates of the sphingolipid pathway. Studies have shown that the increase in ceramide levels modulates the function of neutrophils in terms of cell migration, the generation of ROS, NETs, and bactericidal activities [56–59]. Sphingomyelin and diacylglycerol, intermediates of the sphingolipid pathway are elevated along with sphingomyelin synthase during neutrophil differentiation and regulates infiltration and phagocytic activity [60].

Neutrophils utilize glucose via selective pathways for executing distinct effector functions and prefer glycolysis for phagocytosis; stored glycogen and mitochondrial metabolism are used for phagocytosis and chemotaxis; and NETs formation requires pentose phosphate pathway (PPP) and glycolysis [61]. Neutrophils carryout their effector functions by elevating the glucose uptake through the accumulation of glucose transporter 1 (Glut1) on the cell membrane to regulate phagocytosis, ROS production, and pathogen elimination [62]. Glucose metabolism follows glycolysis to produce ATP and the PPP to generate NADPH molecules. NADPH molecules are further reduced by NADPH oxidase in the neutrophils to produce ROS and NETs formation [63, 64]. Our data showed abundant levels of glucose-6-phosphate (G6P), a glycolytic intermediate in animals upon sepsis indicating the possible generation of high levels of NADPH required for NETs production. The PMA treatment increases the OCR in human neutrophils, and the inhibition of mitochondrial respiration does not affect PMA-induced OCR [65]. PMA-stimulated neutrophils show an increase in the phosphorylation of 6-phosphofructo-2-kinase, the limiting enzyme in glycolysis, and the inhibition of this enzyme decreases both the glycolysis rate and the NOX activity in neutrophils [66]. In a similar line, we observed a significant increase in the OCR upon treating neutrophils with PMA. Subsequently, preliminary analysis in neutrophils isolated from T2D subjects with or without sepsis showed a significant reduction in OCR levels, suggesting defective oxidative burst. This may be a consequence of critically reduced G6P in T2D and T2D + sepsis responsible for an inadequate level of NADPH, which in turn leads to the production of ROS and NETs. However, further mechanistic studies are required to validate these findings.

Neutrophils are equipped with low mitochondrial density. Recent studies have demonstrated that as neutrophils mature, mitochondrial metabolism is reduced and overridden by glycolysis; however, upon stimulation mitochondrial metabolism is reactivated through the TCA cycle [67]. Darroch et al. (2023) using zebra fish models demonstrated enhanced mitochondrial biogenesis in neutrophils during emergency granulopoiesis which increased bactericidal activity [68]. Decreased levels of TCA cycle intermediates, lower mitochondrial membrane potential and aberrations in the spatiotemporal organization of mitochondrial lipidome were prominent in T2D murine models upon inducing sepsis. However, mitochondrial density remained unaltered, suggesting the accumulation of dysfunctional mitochondria in T2D. This prompted us to examine mitophagy levels in neutrophils in T2D conditions. High glucose significantly reduced mitophagy, which may contribute to the accumulation of dysfunctional mitochondria in neutrophils in T2D. Mitophagy receptors are located in both OMM and IMM and spatio-temporal changes in cardiolipins in T2D may be responsible for reduced mitophagy [69]. Molecular dynamics simulations of mitochondrial membranes constructed using lipidomics data suggest structural perturbations of the inner mitochondrial membrane in T2D. These computational findings are consistent with our experimental observations of reduced mitochondrial membrane potential, supporting our hypothesis that mitochondrial aberrations in T2D neutrophils may contribute to reduced response to infections. We even observed fragmentation of the inner mitochondrial membrane when sepsis was mimicked in T2D background. Complementary to this observation, we noticed that neutrophils failed to further enhance mitochondrial membrane potential when sepsis was induced in T2D animals. As observed in animal models, preliminary analysis in human subjects showed a reduction in mitochondrial membrane potential in neutrophils from individuals with T2D. These results suggest hyperglycaemia is associated with mitochondrial dysfunction and accordingly, mitochondria in T2D neutrophils appear unable to maintain optimal function under septic conditions. MD simulations offer valuable mechanistic insights into potential membrane structural changes; the data represent computational predictions parameterized by the experimentally derived lipidomic profiles. As the simulation analysis shows that mitochondrial membrane is ruptured in T2D condition, this observation also suggests that the upstream enzymatic, metabolic, and signalling pathways are also impaired. Hence, comprehensive biochemical analysis may provide deeper insights into mitochondrial dysfunction in neutrophils in T2D and impeded response to infections. Multiple studies have demonstrated different stimuli in infections and inflammation alter mitochondrial functions. For example infections alter mitochondrial homeostasis by elevating mitochondrial ROS, activating NLRP3 inflammasome and IL-1β release [70]. Further, NLRP3-cardiolipin interaction is essential for activation of inflammasome and plays a central role in innate immune responses [71]. Interestingly, our data showed significantly reduced IL-1β levels in T2D upon sepsis. Several studies have shown impaired mitophagy in T2D in multiple tissues and processes such as retinopathy [72], insulin resistance, neurodegeneration [73], hepatic dysfunction [74] and β cell death [75].

We observed that arachidonic acid (AA) and downstream metabolites prostaglandins and leukotrienes were also reduced in T2D animals upon induction of sepsis. Although the influence of AA on neutrophil functions is not known, Tithof et al., (1998) showed that AA stimulates the production of eicosanoid superoxide anion (O_2_^−^) generation in neutrophils [76] and mitochondrial ROS production in sertoli cells [77]. Markworth et al. showed that an increase in the dietary uptake of arachidonic acid (1.5 g/day) by resistance-trained men drastically changed plasma and muscle fatty acid profiles and promoted myogenic gene expression [78]. In rodent model, Zhang et al. showed that intraperitoneal injection of arachidonic acid (150 mg/kg) reduces inflammation in macrophages and increases the survival rate of mice administered with LPS [79]. Arachidonic acid metabolites are actively involved in resolution of inflammation as well. AA-derived lipoxin A4 participates in pausing the neutrophil infiltration, stimulates macrophages to clear the apoptotic cells, reduces leukotriene C4-induced bronchoconstriction in asthmatic subjects [80–84]. Arachidonic acid also exerts tumoricidal properties. AA in its free state reduced the in vitro growth of human cervical carcinoma (HELA) cells and methyl cholanthrene-induced sarcoma cells [85]. Free unesterified AA showed cytotoxic effect on resistant (KB-Ch(R)-8-5) and vincristine-sensitive (KB-3-1) cancer cells in vitro that appeared to be a free-radical dependent process [86]. Arachidonic acid is a primary molecule in producing leukotrienes which plays an important role in driving proinflammatory status. Although, our findings on ex vivo experiments of arachidonic acid showed potentially beneficial effect in T2D + sepsis neutrophil via production of NETs, further studies concerning dosing, delivery method, and potential side effects requires careful validation in T2D models as diabetes is already associated with chronic low-grade inflammation and metabolic changes.

Although further studies are required to understand underlying mechanisms, findings of our study have clinical relevance. Upon sepsis induction in T2D, neutrophils failed to respond due to distinct gene expression patterns in NETs making cells, immuno-metabolic reprogramming and compromised mitochondrial functions. These findings offer potential mechanistic clues relevant to T2D-associated sepsis and may help to design therapeutic strategies aimed at modulating metabolism or restoring mitochondrial function. Arachidonic acid induces mitochondrial ROS and facilitates NETs formation ex vivo which indicates tailoring specific lipids in diet may be further evaluated to improve neutrophil functions in T2D. Excessive formation of NETs contributes to hyper inflammation and subsequent organ damage. In the context of sepsis NETs may be formed due to activated platelets or increased levels of cytokines which may further lead to thrombosis or hyperinflammatory milieu. Understanding these pathways may facilitate in identifying therapeutic strategies in reducing NETs burden. Further, along with specific cytokines, severity of NETs and neutrophil response may be used as diagnostic and prognostic marker in sepsis patients.

However, our study also poses limitations. Neutrophils display distinct transcriptional profiles and phenotype in different tissues. Extramedullary granulopoiesis indicates spleen as source of heterogeneous neutrophils [87]. Ballesteros et al., showed varied transcriptional clusters in lung, blood, bone marrow and spleen infiltrated neutrophils [88]. Wigerbald et al., displayed the existence of four different types of neutrophil subsets based on distinct transcriptional factors [89]. These studies represent the heterogenous behaviour of neutrophils in different tissues. However, our study considered and characterized neutrophils solely from blood and bone marrow. Further, gene editing technologies, stem cell derived neutrophils and single cell (epi)genomics in T2D models may provide deeper mechanistic insights. Key findings from rodent experiments such as (a) proportions of functionally distinct neutrophils; (b) mitochondrial bioenergetics (membrane potential, mitochondrial ROS, mitochondrial density, mitophagy, extracellular flux analysis) and (c) influence of arachidonic acid in restoring NETs in T2D subjects were validated in a small set of human neutrophils. Interestingly, findings observed in rodent models were in line with human neutrophils. However, considering T2D as a complex disorder with respect to (a) control over glycaemic index; (b) duration; (c) response to therapy; (d) BMI; (e) ethnicity, and (f) type of infections where neutrophil (dys)function may vary. Hence, further mechanistic studies are warranted to validate these findings in a larger and statistically powered T2D cohort.

## Supplementary Information

Below is the link to the electronic supplementary material.

Supplementary Material 1

Supplementary Material 2

Supplementary Material 3

Supplementary Material 4

Supplementary Material 5

Supplementary Material 6

Supplementary Material 7

Supplementary Material 8

## Data Availability

All data needed to evaluate the conclusions in the paper are present in the paper and/or the Supplementary Materials. The raw data obtained from RNA sequencing are available in the NCBI Sequence Read Archive (SRA) under the accession number PRJNA1345858. The count file, experimental design file, and the R script used for differential expression analysis (including preprocessing and normalization steps) have been made publicly available on GitHub at: https://github.com/biowizz/T2D.

## References

[1] Mogilenko DA, Sergushichev A, Artyomov MN. Systems immunology approaches to metabolism. Annu Rev Immunol. 2023;41(1):317–42. 37126419 10.1146/annurev-immunol-101220-031513

[2] Hu T, Liu CH, Lei M, Zeng Q, Li L, Tang H, et al. Metabolic regulation of the immune system in health and diseases: mechanisms and interventions. Sig Transduct Target Ther. 2024;9(1):268. 10.1038/s41392-024-01954-6 PMC11461632 39379377

[3] Daryabor G, Atashzar MR, Kabelitz D, Meri S, Kalantar K. The effects of type 2 diabetes mellitus on organ metabolism and the immune system. Front Immunol. 2020;11:1582. 32793223 10.3389/fimmu.2020.01582 PMC7387426

[4] Giacco F, Brownlee M. Oxidative Stress and Diabetic Complications. Schmidt AM, editor. Circulation Research. 2010;107(9):1058–70. 10.1161/CIRCRESAHA.110.223545 PMC2996922 21030723

[5] Roy R, Zayas J, Singh SK, Delgado K, Wood SJ, Mohamed MF, et al. Overriding impaired FPR chemotaxis signaling in diabetic neutrophil stimulates infection control in murine diabetic wound. eLife. 2022;11:e72071. 35112667 10.7554/eLife.72071 PMC8846594

[6] Joshi MB, Ahamed R, Hegde M, Nair AS, Ramachandra L, Satyamoorthy K. Glucose induces metabolic reprogramming in neutrophils during type 2 diabetes to form constitutive extracellular traps and decreased responsiveness to lipopolysaccharides. Biochimica et biophysica acta (BBA). Mol Basis Disease. 2020;1866(12):165940. 10.1016/j.bbadis.2020.165940 32827651

[7] Herrera BS, Hasturk H, Kantarci A, Freire MO, Nguyen O, Kansal S, et al. Impact of Resolvin E1 on Murine Neutrophil Phagocytosis in Type 2 Diabetes. McCormick BA, editor. Infect Immun. 2015;83(2):792–801. 10.1128/IAI.02444-14 PMC4294250 25486994

[8] Gupta S, Maratha A, Siednienko J, Natarajan A, Gajanayake T, Hoashi S, et al. Analysis of inflammatory cytokine and TLR expression levels in type 2 diabetes with complications. Sci Rep. 2017;7(1):7633. 28794498 10.1038/s41598-017-07230-8 PMC5550417

[9] Wong SL, Demers M, Martinod K, Gallant M, Wang Y, Goldfine AB, et al. Diabetes primes neutrophils to undergo NETosis, which impairs wound healing. Nat Med. 2015;21(7):815–9. 26076037 10.1038/nm.3887 PMC4631120

[10] Miyoshi A, Yamada M, Shida H, Nakazawa D, Kusunoki Y, Nakamura A, et al. Circulating neutrophil extracellular trap levels in Well-Controlled type 2 diabetes and pathway involved in their formation induced by High-Dose glucose. Pathobiology. 2016;83(5):243–51. 27189166 10.1159/000444881

[11] Joshi MB, Lad A, Bharath Prasad AS, Balakrishnan A, Ramachandra L, Satyamoorthy K. High glucose modulates IL-6 mediated immune homeostasis through impeding neutrophil extracellular trap formation. FEBS Lett. 2013;587(14):2241–6. 23735697 10.1016/j.febslet.2013.05.053

[12] Joshi MB, Baipadithaya G, Balakrishnan A, Hegde M, Vohra M, Ahamed R, et al. Elevated homocysteine levels in type 2 diabetes induce constitutive neutrophil extracellular traps. Sci Rep. 2016;6(1):36362. 27811985 10.1038/srep36362 PMC5095649

[13] Kuwabara WMT, Yokota CNF, Curi R, Alba-Loureiro TC. Obesity and type 2 diabetes mellitus induce lipopolysaccharide tolerance in rat neutrophils. Sci Rep. 2018;8(1):17534. 30510205 10.1038/s41598-018-35809-2 PMC6277411

[14] Schuetz P, Castro P, Shapiro NI. Diabetes and sepsis: preclinical findings and clinical relevance. Diabetes Care. 2011;34(3):771–8. 21357364 10.2337/dc10-1185 PMC3041224

[15] Ganesh K, Joshi MB. Neutrophil sub-types in maintaining immune homeostasis during steady state, infections and sterile inflammation. Inflamm Res. 2023;72(6):1175–92. 37212866 10.1007/s00011-023-01737-9 PMC10201050

[16] Furze RC, Rankin SM. The role of the bone marrow in neutrophil clearance under homeostatic conditions in the mouse. FASEB J. 2008;22(9):3111–9. 18509199 10.1096/fj.08-109876 PMC2593561

[17] Malengier-Devlies B, Metzemaekers M, Wouters C, Proost P, Matthys P. Neutrophil homeostasis and emergency granulopoiesis: the example of systemic juvenile idiopathic arthritis. Front Immunol. 2021;12:766620. 34966386 10.3389/fimmu.2021.766620 PMC8710701

[18] Ewels PA, Peltzer A, Fillinger S, Patel H, Alneberg J, Wilm A, et al. The nf-core framework for community-curated bioinformatics pipelines. Nat Biotechnol. 2020;38(3):276–8. 32055031 10.1038/s41587-020-0439-x

[19] Tommaso PD, Chatzou M, Floden EW, Barja PP, Palumbo E, Notredame C. Nextflow enables reproducible computational workflows. Nat Biotechnol. 2017;35(4):316–9. 28398311 10.1038/nbt.3820

[20] Andrews S. Babraham Bioinformatics - FastQC A Quality Control tool for High Throughput Sequence Data [Internet]. [cited 2025 Jan 30]. Available from: https://www.bioinformatics.babraham.ac.uk/projects/fastqc/

[21] Ewels P, Magnusson M, Lundin S, Käller M, MultiQC. Summarize analysis results for multiple tools and samples in a single report. Bioinformatics. 2016;32(19):3047–8. 27312411 10.1093/bioinformatics/btw354 PMC5039924

[22] Dobin A, Davis CA, Schlesinger F, Drenkow J, Zaleski C, Jha S, et al. STAR: ultrafast universal RNA-seq aligner. Bioinformatics. 2013;29(1):15–21. 23104886 10.1093/bioinformatics/bts635 PMC3530905

[23] Liao Y, Smyth GK, Shi W. FeatureCounts: an efficient general purpose program for assigning sequence reads to genomic features. Bioinformatics. 2014;30(7):923–30. 24227677 10.1093/bioinformatics/btt656

[24] Robinson MD, McCarthy DJ, Smyth GK. EdgeR: a bioconductor package for differential expression analysis of digital gene expression data. Bioinformatics. 2010;26(1):139–40. 19910308 10.1093/bioinformatics/btp616 PMC2796818

[25] Wassenaar TA, Ingólfsson HI, Böckmann RA, Tieleman DP, Marrink SJ. Computational lipidomics with *insane*: A versatile tool for generating custom membranes for molecular simulations. J Chem Theory Comput. 2015;11(5):2144–55. 26574417 10.1021/acs.jctc.5b00209

[26] Ardail D, Privat JP, Egret-Charlier M, Levrat C, Lerme F, Louisot P. Mitochondrial contact sites. Lipid composition and dynamics. J Biol Chem. 1990;265(31):18797–802. 2172233

[27] Marrink SJ, Risselada HJ, Yefimov S, Tieleman DP, De Vries AH. The MARTINI force field: coarse grained model for biomolecular simulations. J Phys Chem B. 2007;111(27):7812–24. 17569554 10.1021/jp071097f

[28] Van Der Spoel D, Lindahl E, Hess B, Groenhof G, Mark AE, Berendsen HJC. GROMACS: Fast, flexible, and free. J Comput Chem. 2005;26(16):1701–18. 16211538 10.1002/jcc.20291

[29] Bussi G, Donadio D, Parrinello M. Canonical sampling through velocity rescaling. J Chem Phys. 2007;126(1):014101. 17212484 10.1063/1.2408420

[30] Parrinello M, Rahman A. Polymorphic transitions in single crystals: A new molecular dynamics method. J Appl Phys. 1981;52(12):7182–90.

[31] Ban T, Ishihara T, Kohno H, Saita S, Ichimura A, Maenaka K, et al. Molecular basis of selective mitochondrial fusion by heterotypic action between OPA1 and Cardiolipin. Nat Cell Biol. 2017;19(7):856–63. 28628083 10.1038/ncb3560

[32] Zhou Y, Tao W, Shen F, Du W, Xu Z, Liu Z. The emerging role of neutrophil extracellular traps in Arterial, venous and Cancer-Associated thrombosis. Front Cardiovasc Med. 2021;8:786387. 34926629 10.3389/fcvm.2021.786387 PMC8674622

[33] Park J, Wysocki RW, Amoozgar Z, Maiorino L, Fein MR, Jorns J, et al. Cancer cells induce metastasis-supporting neutrophil extracellular DNA traps. Sci Transl Med [Internet]. 2016 Oct 19 [cited 2025 Apr 12];8(361). Available from: https://www.science.org/doi/10.1126/scitranslmed.aag1711 10.1126/scitranslmed.aag1711 PMC5550900 27798263

[34] Pietronigro EC, Della Bianca V, Zenaro E, Constantin G. NETosis in Alzheimer’s Disease. Front Immunol [Internet]. 2017 Mar 2 [cited 2025 Apr 12];8. Available from: http://journal.frontiersin.org/article/10.3389/fimmu.2017.00211/full 10.3389/fimmu.2017.00211 PMC5332471 28303140

[35] Branzk N, Lubojemska A, Hardison SE, Wang Q, Gutierrez MG, Brown GD, et al. Neutrophils sense microbe size and selectively release neutrophil extracellular traps in response to large pathogens. Nat Immunol. 2014;15(11):1017–25. 25217981 10.1038/ni.2987 PMC4236687

[36] Zhang D, Chen G, Manwani D, Mortha A, Xu C, Faith JJ, et al. Neutrophil ageing is regulated by the Microbiome. Nature. 2015;525(7570):528–32. 26374999 10.1038/nature15367 PMC4712631

[37] Kolaczkowska E, Kubes P. Neutrophil recruitment and function in health and inflammation. Nat Rev Immunol. 2013;13(3):159–75. 23435331 10.1038/nri3399

[38] Li Z, Lin Y, Zhang S, Zhou L, Yan G, Wang Y, et al. Emodin regulates neutrophil phenotypes to prevent hypercoagulation and lung carcinogenesis. J Transl Med. 2019;17(1):90. 30885207 10.1186/s12967-019-1838-y PMC6423780

[39] Hacbarth E, Kajdacsy-Balla A. Low density neutrophils in patients with systemic lupus erythematosus, rheumatoid arthritis, and acute rheumatic fever. Arthr Rhuem. 1986;29(11):1334–42. 10.1002/art.1780291105 2430586

[40] Xie X, Shi Q, Wu P, Zhang X, Kambara H, Su J, et al. Single-cell transcriptome profiling reveals neutrophil heterogeneity in homeostasis and infection. Nat Immunol. 2020;21(9):1119–33. 32719519 10.1038/s41590-020-0736-z PMC7442692

[41] Puga I, Cols M, Barra CM, He B, Cassis L, Gentile M, et al. B cell–helper neutrophils stimulate the diversification and production of Immunoglobulin in the marginal zone of the spleen. Nat Immunol. 2012;13(2):170–80. 10.1038/ni.2194 PMC3262910 22197976

[42] Massena S, Christoffersson G, Vågesjö E, Seignez C, Gustafsson K, Binet F, et al. Identification and characterization of VEGF-A–responsive neutrophils expressing CD49d, VEGFR1, and CXCR4 in mice and humans. Blood. 2015;126(17):2016–26. 26286848 10.1182/blood-2015-03-631572 PMC4616235

[43] Liu YJ, Li JP, Han M, Li JX, Ye QW, Lin ST, et al. IFIT1 + neutrophil is a causative factor of immunosuppressive features of poorly cohesive carcinoma (PCC). J Transl Med. 2024;22(1):580. 10.1186/s12967-024-05389-z PMC11188200 38898490

[44] Yipp BG, Petri B, Salina D, Jenne CN, Scott BNV, Zbytnuik LD, et al. Infection-induced NETosis is a dynamic process involving neutrophil multitasking in vivo. Nat Med. 2012;18(9):1386–93. 22922410 10.1038/nm.2847 PMC4529131

[45] Dumont BL, Neagoe PE, Charles E, Villeneuve L, Tardif JC, Räkel A, et al. Low-Density neutrophils contribute to subclinical inflammation in patients with type 2 diabetes. IJMS. 2024;25(3):1674. 38338951 10.3390/ijms25031674 PMC10855851

[46] Lin JC, Siu LK, Fung CP, Tsou HH, Wang JJ, Chen CT, et al. Impaired phagocytosis of capsular serotypes K1 or K2 *Klebsiella pneumoniae* in type 2 diabetes mellitus patients with poor glycemic control. J Clin Endocrinol Metabolism. 2006;91(8):3084–7. 10.1210/jc.2005-2749 16720670

[47] American Diabetes Association. Standards of medical care in Diabetes—2013. Diabetes Care. 2013;36(Supplement1):S11–66. 23264422 10.2337/dc13-S011 PMC3537269

[48] Ayelign B, Negash M, Genetu M, Wondmagegn T, Shibabaw T. Immunological impacts of diabetes on the susceptibility of Mycobacterium tuberculosis. J Immunol Res. 2019;2019:1–8. 10.1155/2019/6196532 PMC6754884 31583258

[49] Chao WC, Yen CL, Wu YH, Chen SY, Hsieh CY, Chang TC, et al. Increased resistin May suppress reactive oxygen species production and inflammasome activation in type 2 diabetic patients with pulmonary tuberculosis infection. Microbes Infect. 2015;17(3):195–204. 25528597 10.1016/j.micinf.2014.11.009

[50] Hair PS, Echague CG, Rohn RD, Krishna NK, Nyalwidhe JO, Cunnion KM. Hyperglycemic conditions inhibit C3-mediated Immunologic control of Staphylococcus aureus. J Transl Med. 2012;10(1):35. 22390383 10.1186/1479-5876-10-35 PMC3328285

[51] Prada-Medina CA, Fukutani KF, Pavan Kumar N, Gil-Santana L, Babu S, Lichtenstein F, et al. Systems immunology of Diabetes-Tuberculosis comorbidity reveals signatures of disease complications. Sci Rep. 2017;7(1):1999. 28515464 10.1038/s41598-017-01767-4 PMC5435727

[52] Moorthy AN, Tan KB, Wang S, Narasaraju T, Chow VT. Effect of High-Fat Diet on the Formation of Pulmonary Neutrophil Extracellular Traps during Influenza Pneumonia in BALB/c Mice. Front Immunol [Internet]. 2016 Aug 2 [cited 2025 Apr 12];7. Available from: http://journal.frontiersin.org/Article/10.3389/fimmu.2016.00289/abstract 10.3389/fimmu.2016.00289 PMC4969943 27531997

[53] Syed SN, Weigert A, Brüne B. Sphingosine kinases are involved in macrophage NLRP3 inflammasome transcriptional induction. IJMS. 2020;21(13):4733. 32630814 10.3390/ijms21134733 PMC7370080

[54] Nakamura T, Abe A, Balazovich KJ, Wu D, Suchard SJ, Boxer LA, et al. Ceramide regulates oxidant release in adherent human neutrophils. J Biol Chem. 1994;269(28):18384–9. 8034585

[55] Zhao X, Yang L, Chang N, Hou L, Zhou X, Dong C, et al. Neutrophil recruitment mediated by sphingosine 1-phosphate (S1P)/S1P receptors during chronic liver injury. Cell Immunol. 2021;359:104243. 33197723 10.1016/j.cellimm.2020.104243

[56] Izawa K, Maehara A, Isobe M, Yasuda Y, Urai M, Hoshino Y, et al. Disrupting ceramide-CD300f interaction prevents septic peritonitis by stimulating neutrophil recruitment. Sci Rep. 2017;7(1):4298. 28655892 10.1038/s41598-017-04647-z PMC5487349

[57] Karandashova S, Kummarapurugu AB, Zheng S, Chalfant CE, Voynow JA. Neutrophil elastase increases airway ceramide levels via upregulation of Serine palmitoyltransferase. Am J Physiology-Lung Cell Mol Physiol. 2018;314(1):L206–14. 10.1152/ajplung.00322.2017 PMC5866429 29025713

[58] Mansfield PJ, Hinkovska-Galcheva V, Carey SS, Shayman JA, Boxer LA. Regulation of polymorphonuclear leukocyte degranulation and oxidant production by ceramide through Inhibition of phospholipase D. Blood. 2002;99(4):1434–41. 11830497 10.1182/blood.v99.4.1434

[59] Meher AK, Spinosa M, Davis JP, Pope N, Laubach VE, Su G, et al. Novel role of IL (Interleukin)-1β in neutrophil extracellular trap formation and abdominal aortic aneurysms. ATVB. 2018;38(4):843–53. 10.1161/ATVBAHA.117.309897 PMC5864548 29472233

[60] Qureshi A, Subathra M, Grey A, Schey K, Del Poeta M, Luberto C. Role of Sphingomyelin Synthase in Controlling the Antimicrobial Activity of Neutrophils against Cryptococcus neoformans. Wang P, editor. PLoS ONE. 2010;5(12):e15587. 10.1371/journal.pone.0015587 PMC3011003 21203393

[61] Injarabian L. Impact of oxygen and glucose differential availability on human neutrophil viability, metabolism and activation. doctoral thesis. 2020.

[62] Li DD, Jawale CV, Zhou C, Lin L, Trevejo-Nunez GJ, Rahman SA, et al. Fungal sensing enhances neutrophil metabolic fitness by regulating antifungal Glut1 activity. Cell Host Microbe. 2022;30(4):530–e5446. 35316647 10.1016/j.chom.2022.02.017 PMC9026661

[63] Azevedo EP, Rochael NC, Guimarães-Costa AB, De Souza-Vieira TS, Ganilho J, Saraiva EM, et al. A metabolic shift toward Pentose phosphate pathway is necessary for amyloid Fibril- and phorbol 12-Myristate 13-Acetate-induced neutrophil extracellular trap (NET) formation. J Biol Chem. 2015;290(36):22174–83. 26198639 10.1074/jbc.M115.640094 PMC4571968

[64] Petty HR, Kindzelskii AL, Chaiworapongsa T, Petty AR, Romero R. Oxidant release is dramatically increased by elevated glucose concentrations in neutrophils from pregnant women. J Maternal-Fetal Neonatal Med. 2005;18(6):397–404. 10.1080/14767050500361679 16390806

[65] Chacko BK, Kramer PA, Ravi S, Johnson MS, Hardy RW, Ballinger SW, et al. Methods for defining distinct bioenergetic profiles in platelets, lymphocytes, monocytes, and neutrophils, and the oxidative burst from human blood. Lab Invest. 2013;93(6):690–700. 23528848 10.1038/labinvest.2013.53 PMC3674307

[66] Baillet A, Hograindleur M, El Benna J, Grichine A, Berthier S, Morel F, et al. Unexpected function of the phagocyte NADPH oxidase in supporting hyperglycolysis in stimulated neutrophils: key role of 6-phosphofructo‐2‐kinase. FASEB J. 2017;31(2):663–73. 27799347 10.1096/fj.201600720R

[67] Lika J, Votava JA, Datta R, Kralovec AM, Smith FM, Huttenlocher A, et al. Mitochondrial metabolism is rapidly re-activated in mature neutrophils to support stimulation-induced response [Internet]. Immunology; 2025 [cited 2025 Apr 12]. Available from: http://biorxiv.org/lookup/doi/10.1101/2025.02.03.636312 10.3389/fimmu.2025.1572927 PMC12066771 40356902

[68] Darroch H, Keerthisinghe P, Sung YJ, Rolland L, Prankerd-Gough A, Crosier PS, et al. Infection-experienced HSPCs protect against infections by generating neutrophils with enhanced mitochondrial bactericidal activity. Sci Adv. 2023;9(36):eadf9904. 37672586 10.1126/sciadv.adf9904 PMC10482338

[69] Yoo SM, Jung YK. A Molecular Approach to Mitophagy and Mitochondrial Dynamics. 10.14348/molcells.2018.2277 PMC5792708 29370689

[70] Nakahira K, Haspel JA, Rathinam VAK, Lee SJ, Dolinay T, Lam HC, et al. Autophagy proteins regulate innate immune responses by inhibiting the release of mitochondrial DNA mediated by the NALP3 inflammasome. Nat Immunol. 2011;12(3):222–30. 21151103 10.1038/ni.1980 PMC3079381

[71] Akbal A, Dernst A, Lovotti M, Mangan MSJ, McManus RM, Latz E. How location and cellular signaling combine to activate the NLRP3 inflammasome. Cell Mol Immunol. 2022;19(11):1201–14. 36127465 10.1038/s41423-022-00922-w PMC9622870

[72] Zhou P, Xie W, Meng X, Zhai Y, Dong X, Zhang X, Correction: Zhou P, et al. Notoginsenoside R1 Ameliorates Diabetic Retinopathy through PINK1-Dependent Activation of Mitophagy. Cells, 2019, 8, 213. Cells. 2020;9(2):450. 10.3390/cells9020450 PMC7072801 32079148

[73] Scheele C, Nielsen AR, Walden TB, Sewell DA, Fischer CP, Brogan RJ, et al. Altered regulation of the PINK1 locus: a link between type 2 diabetes and neurodegeneration? FASEB J. 2007;21(13):3653–65. 17567565 10.1096/fj.07-8520com

[74] Vanhorebeek I, De Vos R, Mesotten D, Wouters PJ, De Wolf-Peeters C, Van Den Berghe G. Protection of hepatocyte mitochondrial ultrastructure and function by strict blood glucose control with insulin in critically ill patients. Lancet. 2005;365(9453):53–9. 15639679 10.1016/S0140-6736(04)17665-4

[75] Sidarala V, Pearson GL, Parekh VS, Thompson B, Christen L, Gingerich MA, et al. Mitophagy protects β cells from inflammatory damage in diabetes. JCI Insight. 2020;5(24):e141138. 33232298 10.1172/jci.insight.141138 PMC7819751

[76] Tithof PK, Peters-Golden M, Ganey PE. Distinct phospholipases A2 regulate the release of arachidonic acid for eicosanoid production and superoxide anion generation in neutrophils. J Immunol. 1998;160(2):953–60. 9551934

[77] Hu Y, Luo NJ, Gan L, Xue HY, Luo KY, Zhang JJ, et al. Heat stress upregulates arachidonic acid to trigger autophagy in Sertoli cells via dysfunctional mitochondrial respiratory chain function. J Transl Med. 2024;22(1):501. 38797842 10.1186/s12967-024-05182-y PMC11129461

[78] Markworth JF, Mitchell CJ, D’Souza RF, Aasen KMM, Durainayagam BR, Mitchell SM, et al. Arachidonic acid supplementation modulates blood and skeletal muscle lipid profile with no effect on basal inflammation in resistance exercise trained men. Prostaglandins Leukot Essent Fatty Acids. 2018;128:74–86. 29413364 10.1016/j.plefa.2017.12.003

[79] Zhang Y, Chen H, Zhang W, Cai Y, Shan P, Wu D, et al. Arachidonic acid inhibits inflammatory responses by binding to myeloid differentiation factor-2 (MD2) and preventing MD2/toll-like receptor 4 signaling activation. Biochimica et biophysica acta (BBA). Mol Basis Disease. 2020;1866(5):165683. 10.1016/j.bbadis.2020.165683 31953218

[80] Serhan CN. Pro-resolving lipid mediators are leads for resolution physiology. Nature. 2014;510(7503):92–101. 24899309 10.1038/nature13479 PMC4263681

[81] Serhan CN, Chiang N, Dalli J. The resolution code of acute inflammation: novel pro-resolving lipid mediators in resolution. Semin Immunol. 2015;27(3):200–15. 25857211 10.1016/j.smim.2015.03.004 PMC4515371

[82] Börgeson E, McGillicuddy FC, Harford KA, Corrigan N, Higgins DF, Maderna P, et al. Lipoxin A_4_ attenuates adipose inflammation. FASEB J. 2012;26(10):4287–94. 22700871 10.1096/fj.12-208249

[83] Reis MB, Pereira PAT, Caetano GF, Leite MN, Galvão AF, Paula-Silva FWG, et al. Lipoxin A4 encapsulated in PLGA microparticles accelerates wound healing of skin ulcers. Diaz BL, editor. PLoS ONE. 2017;12(7):e0182381. 10.1371/journal.pone.0182381 PMC5533323 28753648

[84] Barnig C, Levy BD. Lipoxin A_4_: a new direction in asthma therapy? Expert Rev Clin Immunol. 2013;9(6):491–3. 23730877 10.1586/eci.13.36

[85] Sagar PS, Das UN. Cytotoxic action of cis-unsaturated fatty acids on human cervical carcinoma (HeLa) cells in vitro. Prostaglandins Leukot Essent Fatty Acids. 1995;53(4):287–99. 8577783 10.1016/0952-3278(95)90129-9

[86] Das UN, Madhavi N. Effect of polyunsaturated fatty acids on drug-sensitive and resistant tumor cells in vitro. Lipids Health Dis. 2011;10(1):159. 21917129 10.1186/1476-511X-10-159 PMC3180408

[87] Guo R. New insights on extramedullary granulopoiesis and neutrophil heterogeneity in the spleen and its importance in disease. 10.1093/jleuko/qiae220 39514106

[88] Ballesteros I, Rubio-Ponce A, Genua M, Lusito E, Kwok I, Fernández-Calvo G, et al. Co-option of neutrophil fates by tissue environments. Cell. 2020;183(5):1282–e129718. 33098771 10.1016/j.cell.2020.10.003

[89] Wigerblad G, Cao Q, Brooks S, Naz F, Gadkari M, Jiang K, et al. Single-Cell analysis reveals the range of transcriptional States of Circulating human neutrophils. J Immunol. 2022;209(4):772–82. 35858733 10.4049/jimmunol.2200154 PMC9712146

