# Supplementary material for: Immuno-metabolic dysregulation in type 2 diabetes is associated with altered neutrophil functional plasticity, mitochondrial dysfunction, and compromised responses in sepsis
Source: J Transl Med. 2026 Jan 10;24:156. doi: 10.1186/s12967-026-07696-z (PMC12882159; doi:10.1186/s12967-026-07696-z)
Supplement: Supplementary file 1 — Supplementary Material 1 [file 12967_2026_7696_MOESM1_ESM.docx]

**Immuno-metabolic changes in Type 2 Diabetes regulate functional plasticity and mitochondrial dysfunction in neutrophils leading to limited response in sepsis.**

Kailash Ganesh *et al*

* Dr Manjunath B Joshi. Email: [*Manjunath.joshi@manipal.edu*](mailto:Manjunath.joshi@manipal.edu)


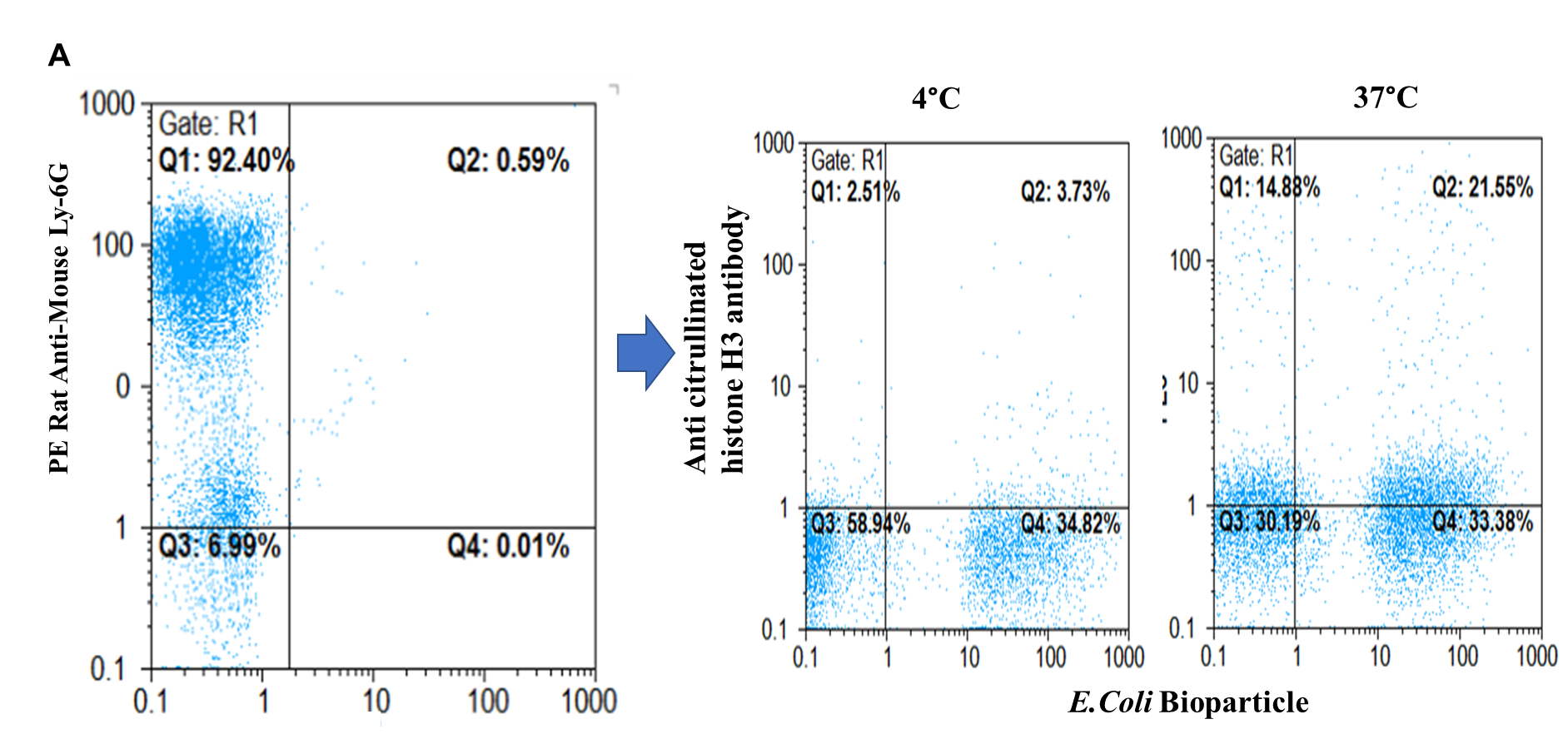


**SF1A:** Peripheral blood neutrophils isolated from control animals were tagged with Ly6G and treated with *E. coli* bioparticle (1:10) at 4 ° C and 37 ° C. Washed cells are stained with Anti citrullinated histone H3 antibody and secondary antibody conjugated with AlexaFluor633


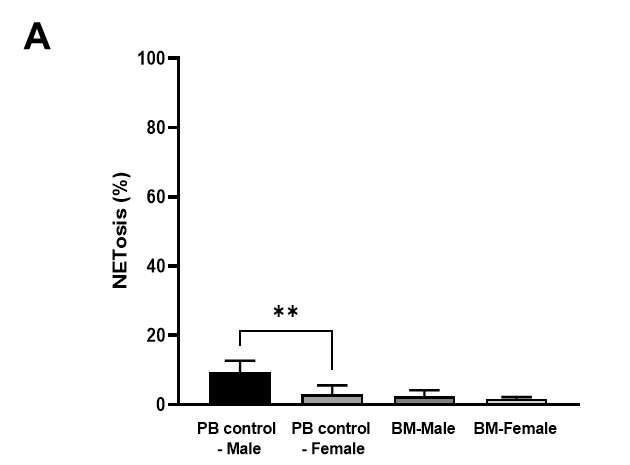


**SF2A:** Peripheral blood and BM neutrophils isolated from healthy male and female mice were stained with Anti citrullinated histone H3 antibody and secondary antibody conjugated with AlexaFluor633. Data is represented bar graph. Bars represent the mean + SD and, * = p<0.01, ** = p<0.001, *** = p<0.0001.


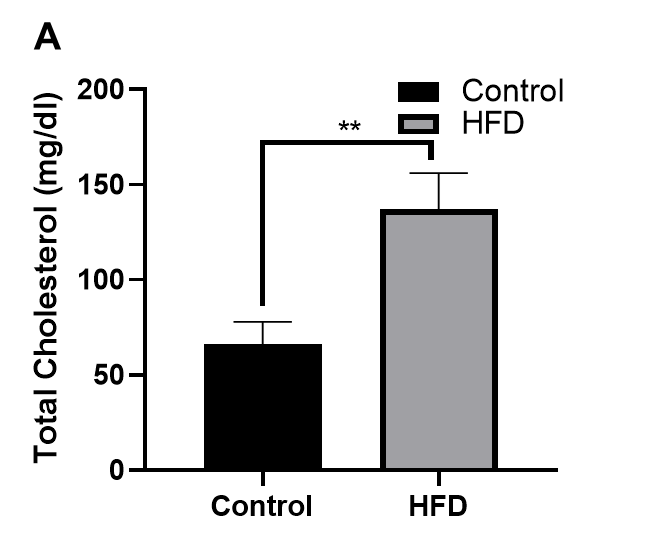


**SF3A:** Plasma collected from control and HFD mice were analysed for Total cholesterol levels using autoanalyzer. Data is represented bar graph. Bars represent the mean + SD and, * = p<0.01, ** = p<0.001, *** = p<0.0001.


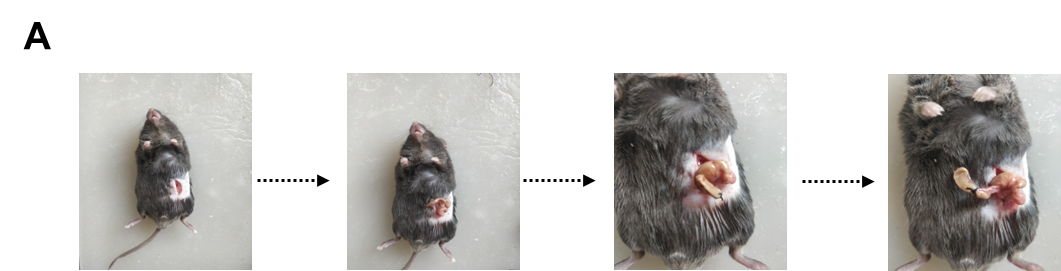


**SF4A:** Cecal ligation and puncture survival surgery procedure to induce sepsis.


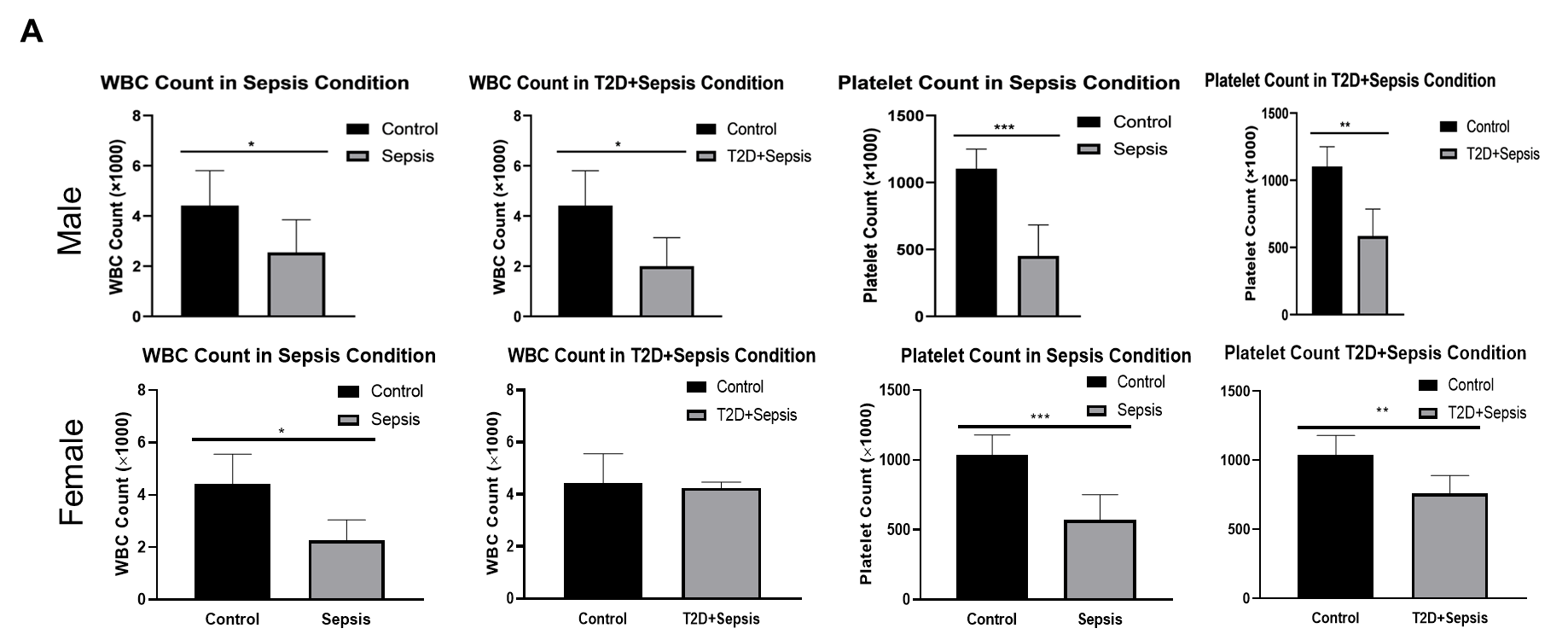


**SF5A:** Blood collected from sepsis induced male and female mice were analysed for WBC count and platelet count using autoanalyzer. Data is represented bar graph. Bars represent the mean + SD and, * = p<0.01, ** = p<0.001, *** = p<0.0001.


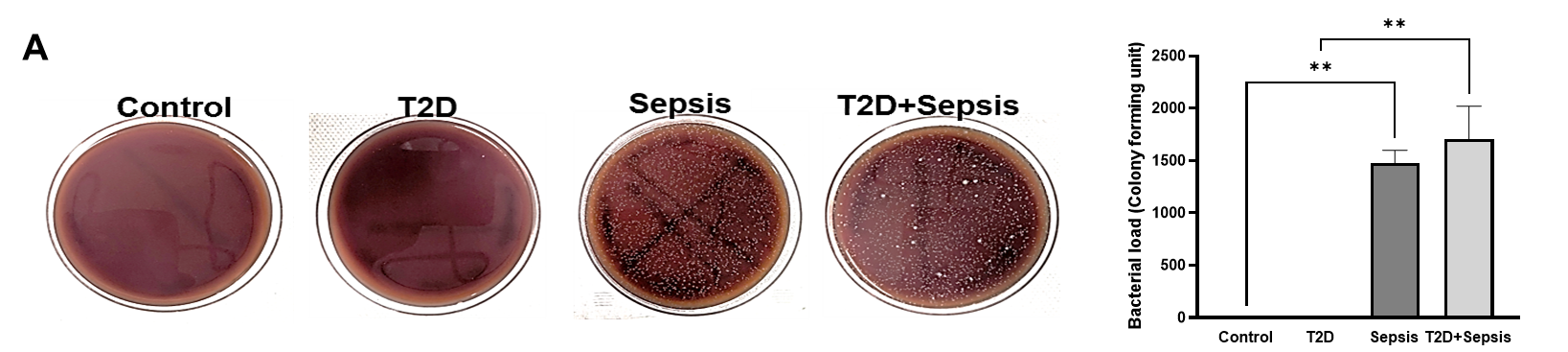


**SF6A:** Blood collected from control, T2D, sepsis and T2D+sepsis were mixed with distilled water at the ratio of 1:1000 was platted on blood agar plate for 24 hours. Colonies formed are represented in bar graph. Bars represent the mean + SD and, * = p<0.01, ** = p<0.001, *** = p<0.0001.


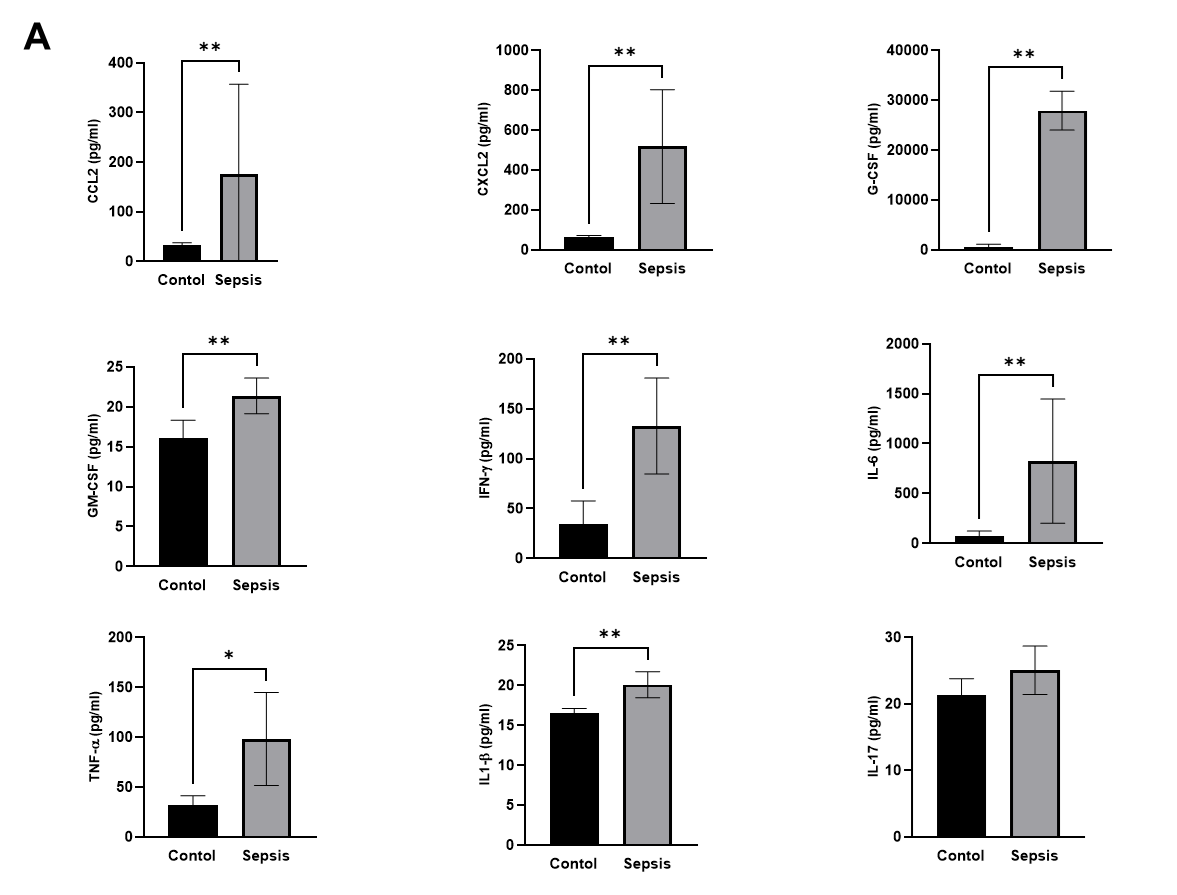


**SF7A:** Cytokines were measured using multiplex ELISA in the plasma of healthy and sepsis mice. Data is represented bar graph. Bars represent the mean + SD and, * = p<0.01, ** = p<0.001, *** = p<0.0001.


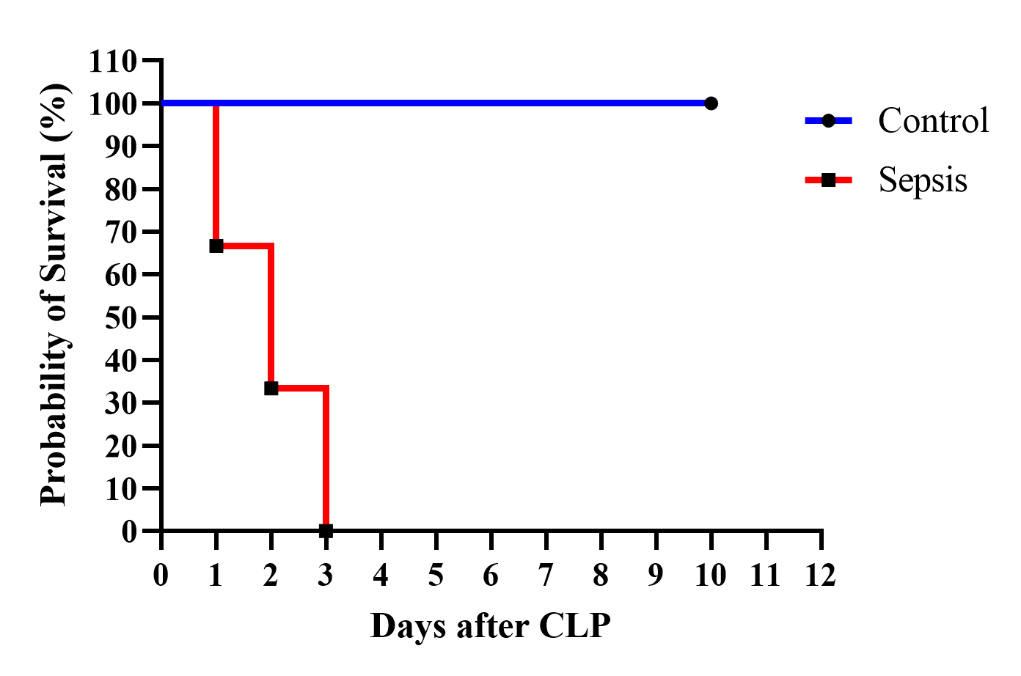


**A**

***

**SF8A:** Mantel-Cox survival curves of sepsis mice (n=6/group) over the period of 12 days. P value = 0.0015.


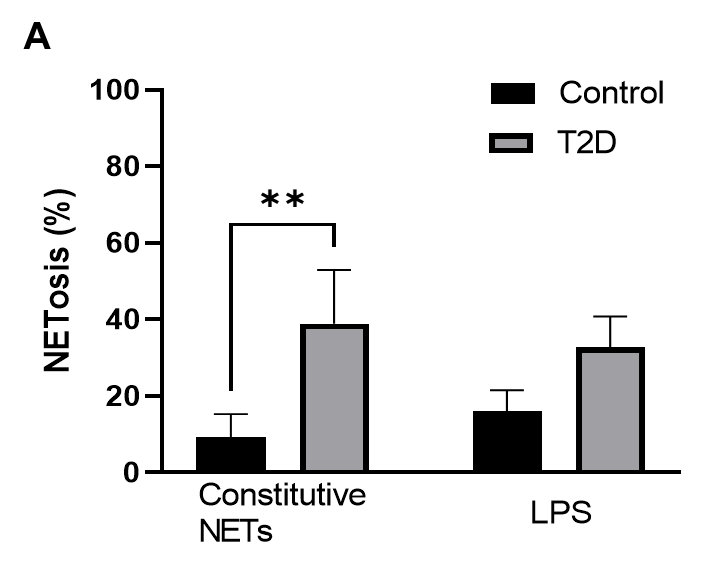


**SF9A:** BM neutrophils isolated from healthy and T2D male mice were treated with LPS for 30 mins and stained with Anti citrullinated histone H3 antibody and secondary antibody conjugated with AlexaFluor633. Data is represented bar graph. Bars represent the mean + SD and, * = p<0.01, ** = p<0.001, *** = p<0.0001.


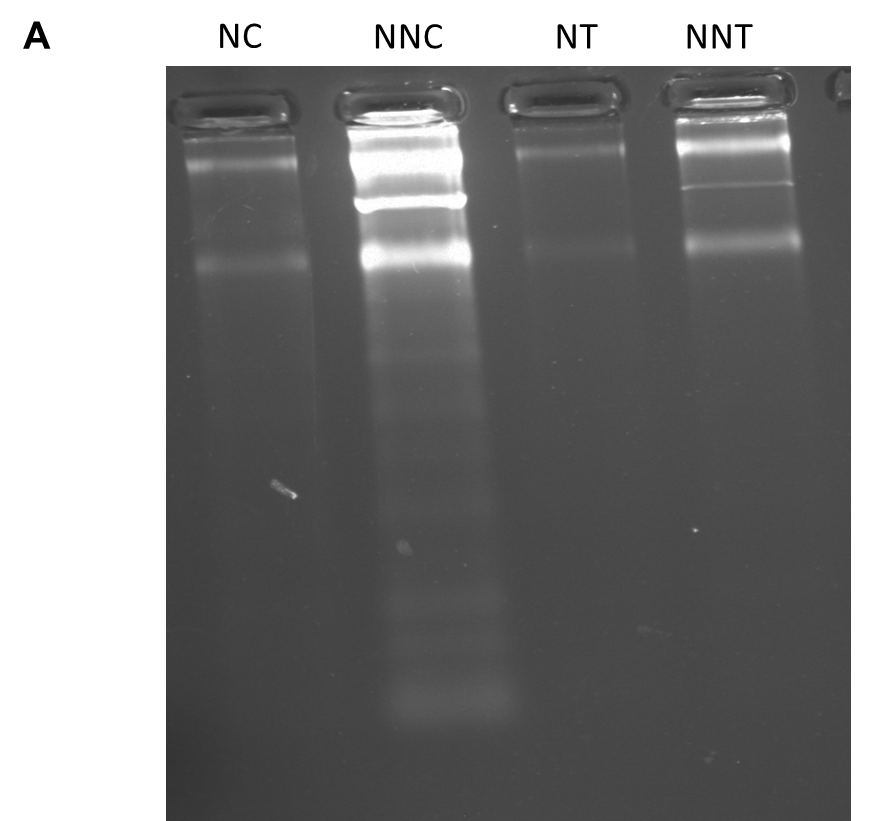


**SF10A:** RNA isolated from NNF and NF of both control and T2D peripheral blood neutrophils were ran on agarose gel for confirmation.


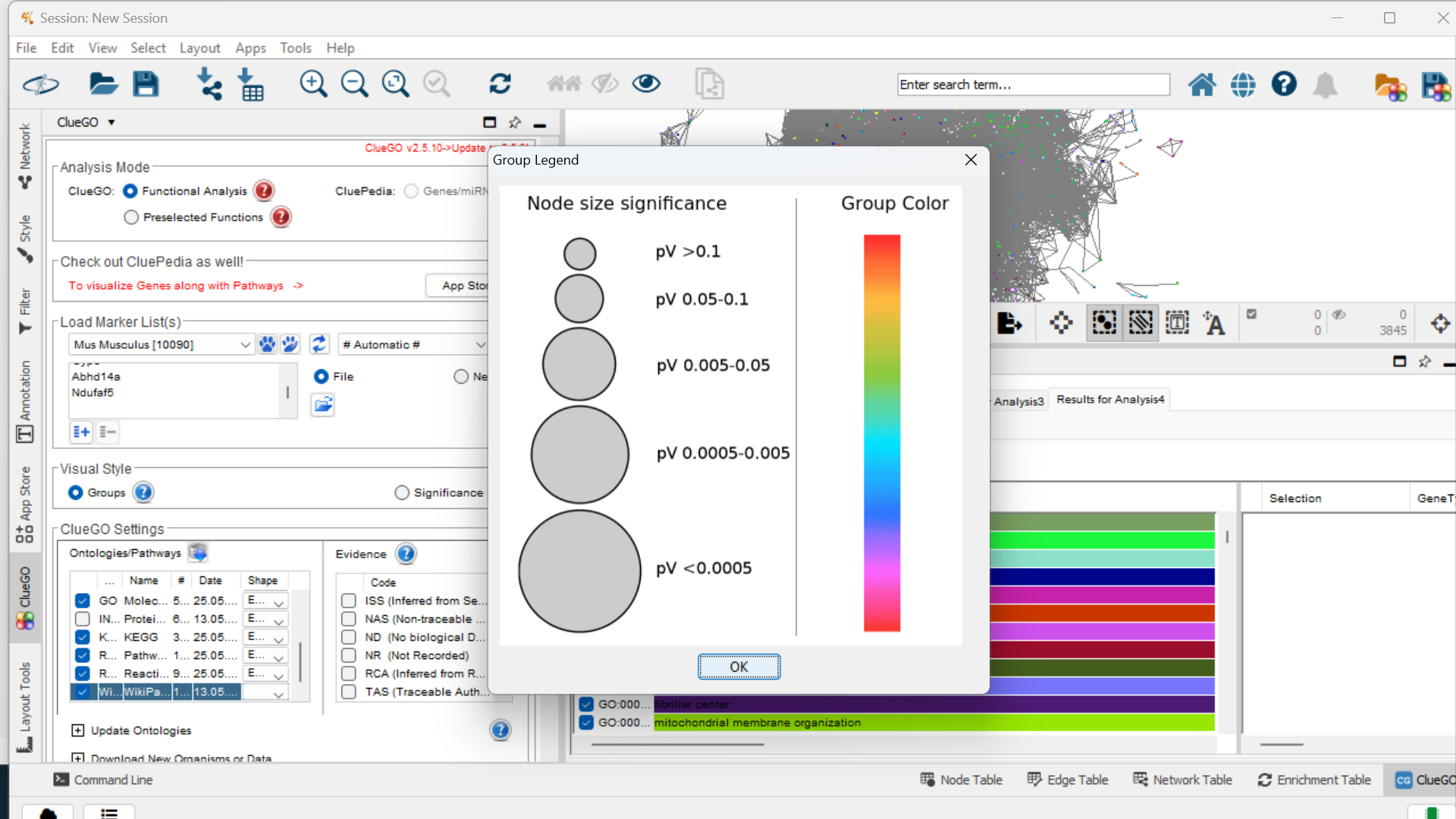


**A**

**SF11A:** Representative p value for the size and colour of the nodes.


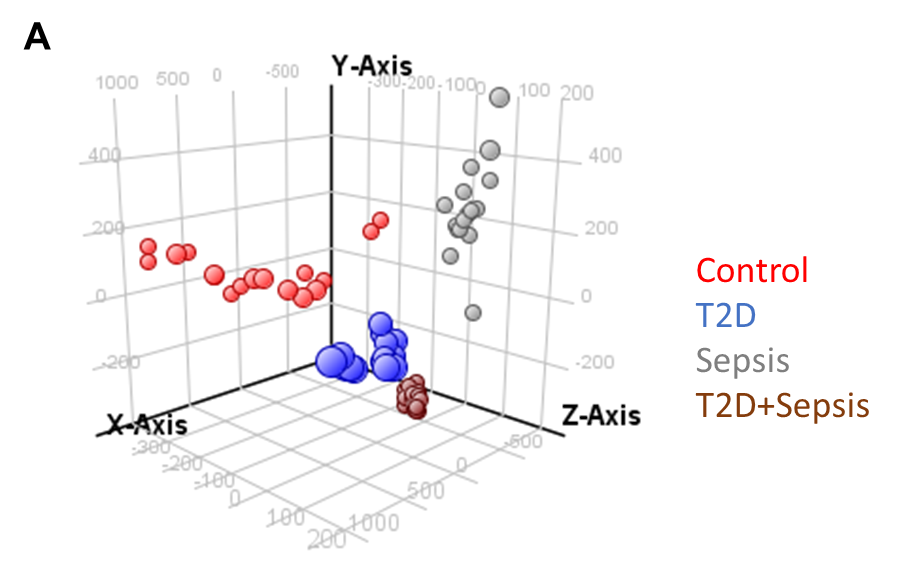


**SF12A:** PCA plot of metabolic runs of control, T2D, sepsis and T2D+sepsis.


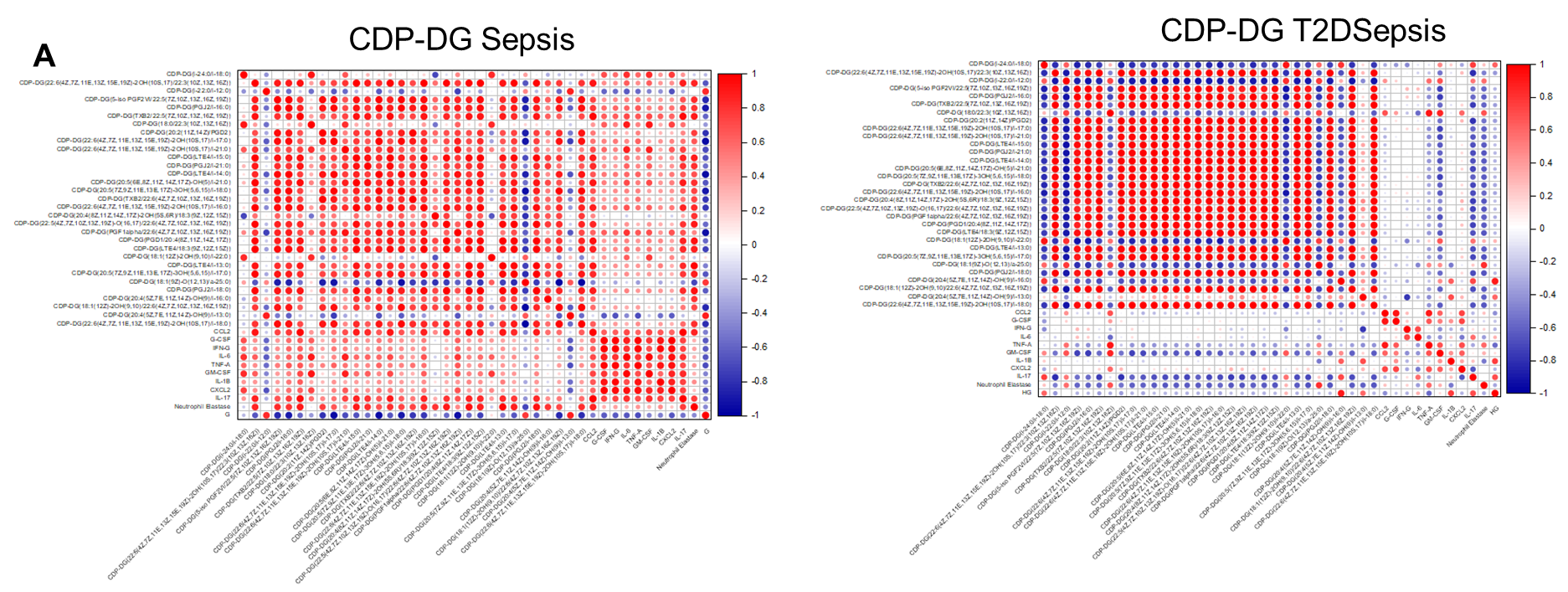


**SF13A:** Pearson’s correlation analysis was performed using abundance of CDP-DG’s and cytokines from sepsis and T2D+ sepsis animals. Colour intensity from white to red indicates increase in the association whereas white to blue indicates negative association between metabolites and cytokines.


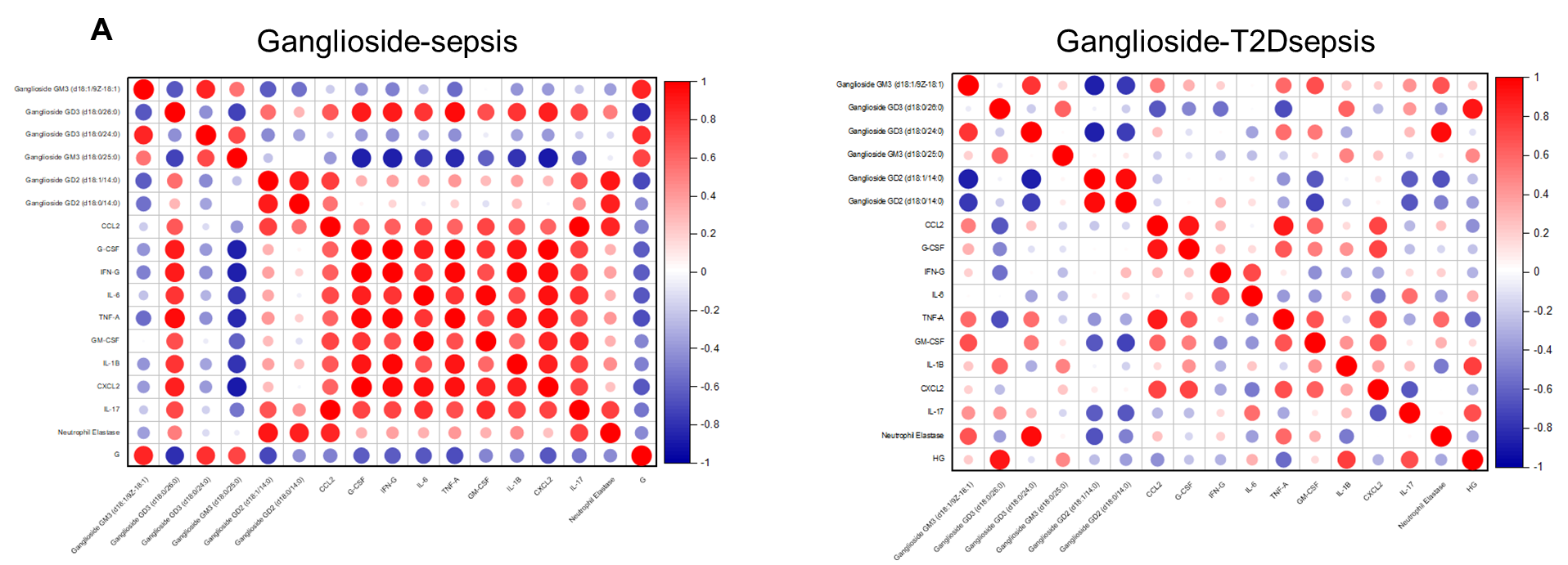


**SF14A:** Pearson’s correlation analysis was performed using abundance of gangliosides and cytokines from sepsis and T2D+ sepsis animals. Colour intensity from white to red indicates increase in the association whereas white to blue indicates negative association between metabolites and cytokines.


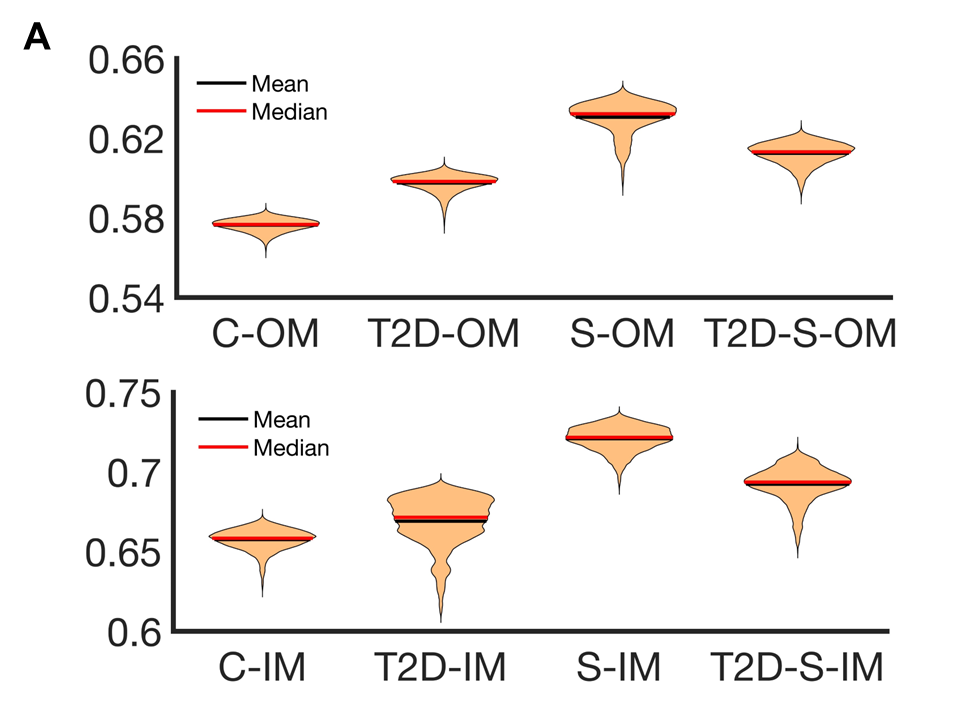


**SF15A**: The area per lipid (considering all the lipids) for all the systems is obtained from the concatenated trajectory of 6 µs by combining three replicas of each system.


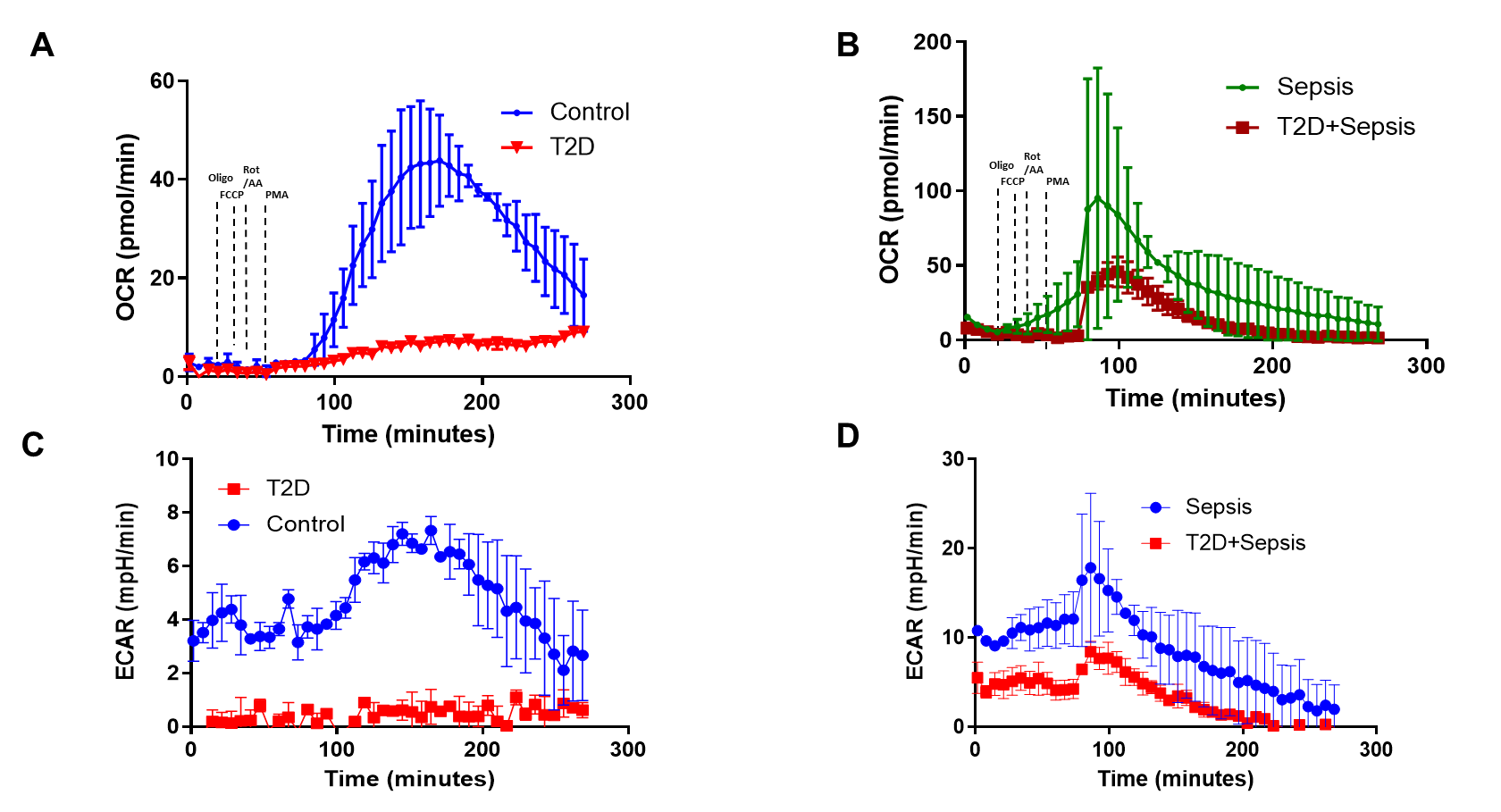


**SF16:** Extracellular flux analysis was performed in neutrophils isolated from healthy and T2D subjects. Approximately 40,000 cells/well were seeded in poly-Lysine coated plates and cultured in RPMI without serum followed by recording of OCR **A)** Healthy & T2D, **B)** T2D+sepsis subject, extracellular acidification rate **C)** healthy & T2D, **D)** sepsis and T2D+sepsis subject.


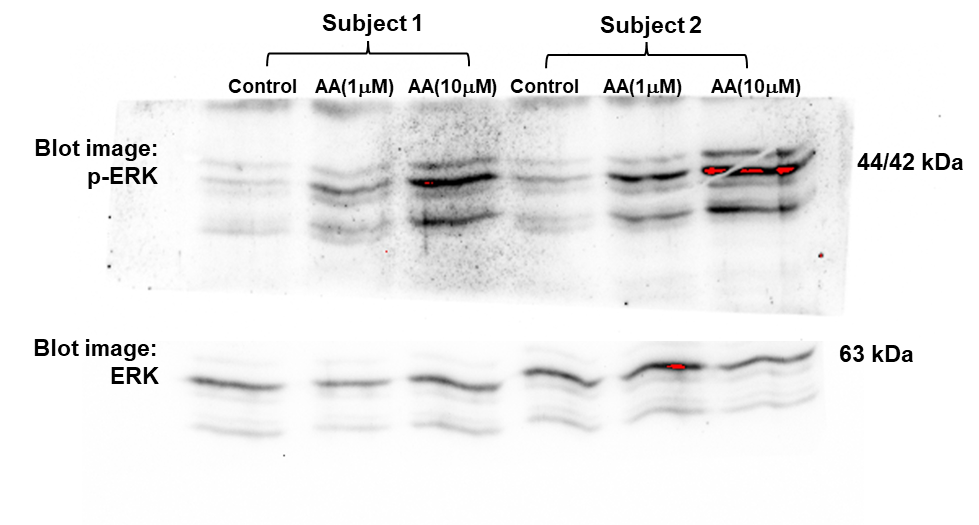


**SF17:** Raw blot image of two samples stained for phosphor-ERK and total-ERK.

**Supplementary Table 1:** Detailed list of genes involved in the pathways, biological process and molecular functions.

| **Term** | **Overlap** | **P-value** | **Genes** |
| --- | --- | --- | --- |
| **NF vs NNF Upregulated genes in control - Pathways** | | | |
| RND1 GTPase Cycle | 7/42 | 0.00016514 | FAM135A;VANGL2;DST;MUC13;FRS3;ARHGAP35;EPHA2 |
| Classical Antibody-Mediated Complement Activation | 3/6 | 0.000429655 | C1QB;C1QA;C1QC |
| Chemokine Receptors Bind Chemokines | 7/56 | 0.001007567 | CX3CR1;CXCL9;CCL5;CCL2;CCR7;CCR5;CCR3 |
| Interleukin-7 Signaling | 4/25 | 0.005098503 | PIK3R3;RAG2;IL7R;RAG1 |
| **NF vs NNF Upregulated genes in control – Biological Process** | | | |
| Histone Methylation | 4/9 | 7.28897E-05 | FBL;PRMT5;SMYD2;SMYD3 |
| Positive Regulation Of Calcium Ion Transport | 6/29 | 0.000140383 | CACNB2;TRPC6;CCL5;PDGFB;CCL2;FFAR1 |
| Cellular Defense Response | 7/48 | 0.000389008 | CX3CR1;NCR1;CXCL9;FCMR;KLRC2;CCR5;CCR3 |
| Peptidyl-Arginine Modification | 3/6 | 0.000429655 | PRMT5;PRMT7;NDUFAF5 |
| Enzyme-Linked Receptor Protein Signaling Pathway | 11/124 | 0.000835276 | BLK;CD4;CD8A;FLT3;NPR2;CD7;IL31RA;CD3E;GFRA2;PTPRF;EPHA2 |
| Regulation Of Phosphatidylinositol 3-Kinase Activity | 5/34 | 0.002574564 | FLT3;CD19;PDGFB;PIK3R3;CCR7 |
| Granulocyte Activation | 3/12 | 0.004159586 | CCL5;CTSG;PRG3 |
| Cellular Response To Lipopolysaccharide | 9/124 | 0.009032606 | CX3CR1;CXCL9;CCL5;TNIP3;CD180;CCL2;CTSG;NR1D1;CCR5 |
| Neutrophil Activation | 3/18 | 0.013594006 | CCL5;CTSG;PRG3 |
| Cytoskeleton Organization | 8/111 | 0.013992687 | NCKIPSD;CECR2;DST;CCL2;WDPCP;THY1;APOE;PLD2 |
| **NF vs NNF Upregulated genes in control – Molecular function** | | | |
| Cytokine Receptor Activity | 10/76 | 5.66974E-05 | CX3CR1;CD4;FLT3;IL2RB;IL31RA;GFRA1;CCR5;IL7R;GFRA2;CCR3 |
| Phospholipase C Activity | 3/23 | 0.026573944 | PLCL1;CCL5;CCR5 |
| Voltage-Gated Calcium Channel Activity | 4/47 | 0.044176539 | CACNA1I;CACNB2;NCS1;CACNA1A |
| **NF vs NNF Upregulated genes in T2D – Pathways** | | | |
| Complement Cascade | 4/55 | 0.012696487 | C1QB;CR2;CD19;C1QC |
| IL-6-type Cytokine Receptor Ligand Interactions | 2/17 | 0.031177675 | CNTF;LIFR |
| **NF vs NNF Downregulated genes in T2D – Pathways** | | | |
| Extracellular Matrix Organization | 5/291 | 0.026501954 | LAMA5;ITGA3;MMP2;LOXL4;CAPN3 |
| **NF vs NNF Upnregulated genes in T2D – Biological process** | | | |
| Enzyme-Linked Receptor Protein Signaling Pathway | 13/124 | 1.3592E-07 | BLK;DDR1;FLT3;NPR2;LIFR;MST1R;CD3E;GFRA2;CD4;CD8A;AXL;EPHA2;FGFR1 |
| Positive Regulation Of Kinase Activity | 9/104 | 5.51788E-05 | DDR1;CD4;FLT3;AXL;CCR7;MST1R;AXIN2;EPHA2;FGFR1 |
| Lymphocyte Mediated Immunity | 3/16 | 0.002106925 | CR2;KLRC2;CD8A |
| Cellular Response To Cytokine Stimulus | 12/308 | 0.005215458 | MAPK11;CD4;AXL;FLT3;NPR2;LEF1;IL2RB;LEPR;IL5RA;XCL1;CCR7;LIFR |
| **NF vs NNF Downregulated genes in T2D – Biological process** | | | |
| Positive Regulation Of Cell Communication | 2/30 | 0.012835922 | RIMS3;ANK3 |
| Extracellular Matrix Organization | 4/176 | 0.018829475 | MMP2;HAS3;LOXL4;NDNF |
| **NF vs NNF Upregulated genes in T2D – Molecular functions** | | | |
| Voltage-Gated Calcium Channel Activity | 6/47 | 0.000115163 | CACNA1I;CACNA2D1;SCN8A;NCS1;CACNA1B;CACNA1G |
| Cytokine Receptor Activity | 7/76 | 0.000252898 | CD4;FLT3;IL2RB;LEPR;IL5RA;LIFR;GFRA2 |
| **NF - control vs NF - T2D Upregulated genes – Biological process** | | | |
| Extracellular Matrix Organization | 6/176 | 0.003549308 | COL17A1;COL1A1;POSTN;ADAMTS14;COL1A2;COL4A6 |
| **NF - control vs NF - T2D Upregulated genes – Biological process** | | | |
| High Voltage-Gated Calcium Channel Activity | 5/23 | 1.10554E-06 | CACNB1;SCN8A;CACNA1B;CACNA1E;CACNA1G |

**Supplementary Table 8:** Description of lipid composition considered in the MD simulations.

*, ** - concentrations are condition dependent


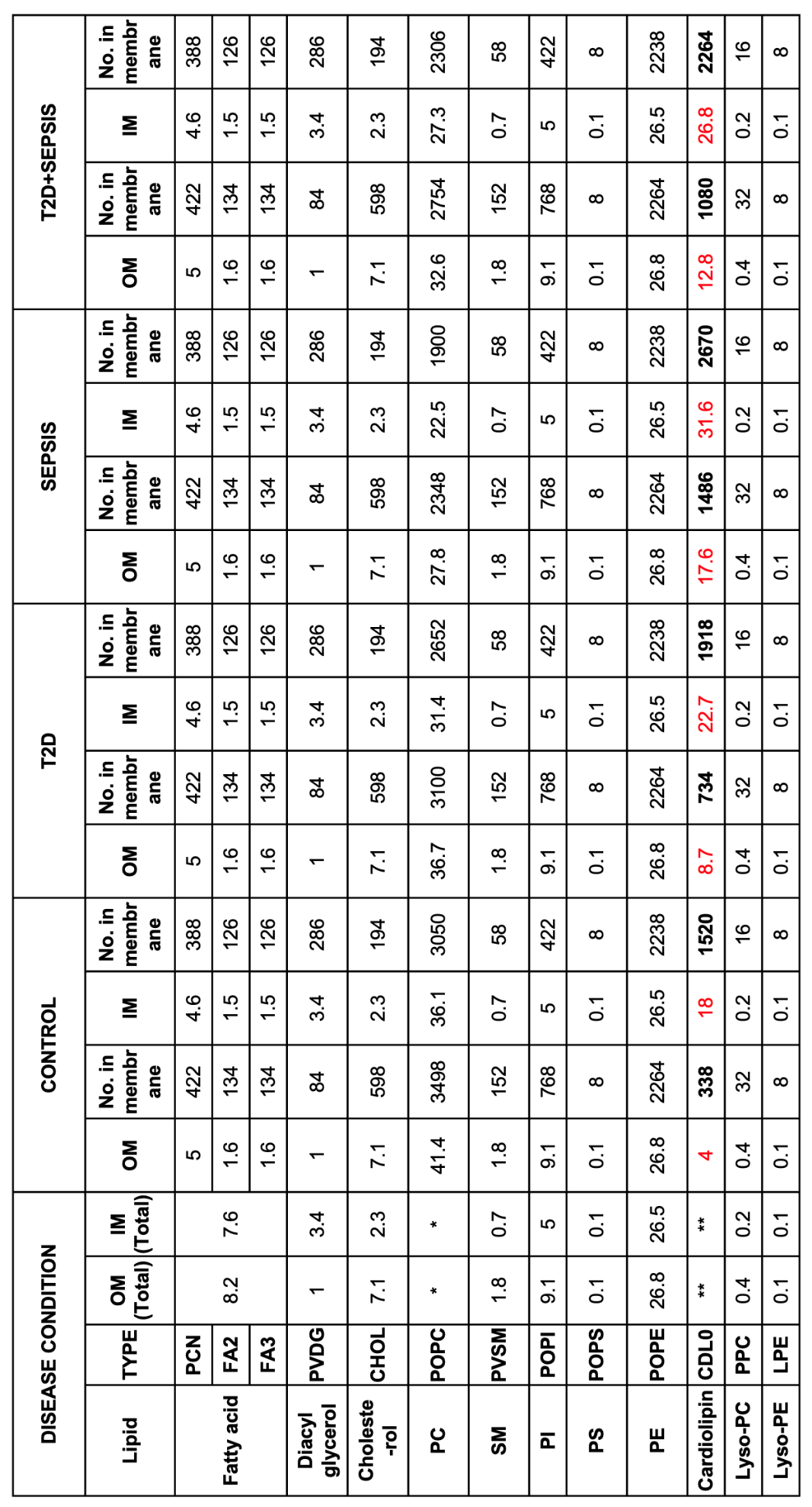


Statistical details:

A priori power calculations were performed using G*Power (v3.1.9.7) for the future studies. For two-sample comparisons (two-tailed t-test, α = 0.05), we computed required sample sizes for Cohen’s d = 0.5, 0.6 and 0.8 at 90% power. The resulting sample sizes per group are

1. d = 0.5 corresponds to 86 samples/group
2. d = 0.6 corresponds to 60 samples/group
3. d = 0.7 corresponds to 44 samples/group
4. d = 0.8 corresponds to 34 samples/group
